# Impact of Synergy Partner Cel7B on Cel7A Binding Rates: Insights from Single-Molecule Data

**DOI:** 10.1021/acs.jpcb.3c05697

**Published:** 2024-01-16

**Authors:** Aimilia Nousi, Gustavo Avelar Molina, Corinna Schiano-di-Cola, Trine Holst Sørensen, Kim Borch, Jonas N. Pedersen, Peter Westh, Rodolphe Marie

**Affiliations:** † Department of Health Technology, Technical University of Denmark, 2800 Kongens Lyngby, Denmark; ‡ Department of Biotechnology and Biomedicine, Technical University of Denmark, 2800 Kongens Lyngby, Denmark; § Novozymes A/S, Krogshøjvej 36, 2880 Bagsværd, Denmark; ∥ The Novo Nordisk Foundation Center for Biosustainability, Technical University of Denmark, 2800 Kongens Lyngby, Denmark

## Abstract

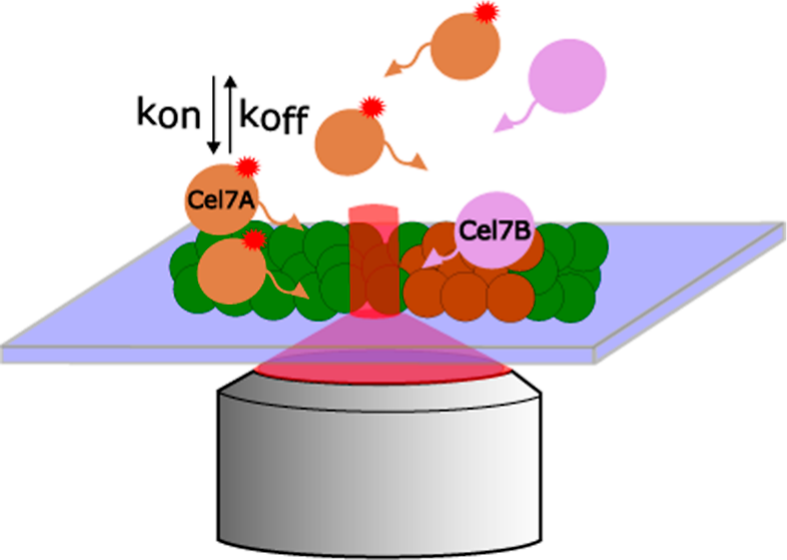

Enzymatic degradation of cellulosic biomass is a well-established route for the sustainable production of biofuels, chemicals, and materials. A strategy employed by nature and industry to achieve an efficient degradation of cellulose is that cellobiohydrolases (or exocellulases), such as Cel7A, work synergistically with endoglucanases, such as Cel7B, to achieve the complete degradation of cellulose. However, a complete mechanistic understanding of this exo–endo synergy is still lacking. Here, we used single-molecule fluorescence microscopy to quantify the binding kinetics of Cel7A on cellulose when it is acting alone on the cellulose fibrils and in the presence of its synergy partner, the endoglucanase Cel7B. To this end, we used a fluorescently tagged Cel7A and studied its binding in the presence of the unlabeled Cel7B. This provided the single-molecule data necessary for the estimation of the rate constants of association *k*_ON_ and dissociation *k*_OFF_ of Cel7A for the substrate. We show that the presence of Cel7B does not impact the dissociation rate constant, *k*_OFF_. But, the association rate of Cel7A decreases by a factor of 2 when Cel7B is present at a molar proportion of 10:1. This ratio has previously been shown to lead to synergy. This decrease in association rate is observed in a wide range of total enzyme concentrations, from sub nM to μM concentrations. This decrease in *k*_ON_ is consistent with the formation of cellulase clusters recently observed by others using atomic force microscopy.

## Introduction

Cellulose is a complex carbohydrate and the most abundant biopolymer on earth.^1^ A cellulose chain consists of thousands of glucose molecules linked together by strong glycosidic bonds. These chains are densely packed to form strong and rigid microfibrils stabilized by intermolecular forces. Due to its high availability, biodegradability, and physical and mechanical properties, cellulose is used in many areas such as paper and textiles production, and it is also studied for biomedical and biotechnological applications.^1,2^ Most importantly, however, the natural abundance of cellulose, as well as its intrinsic renewability, makes it a potential alternative for a sustainable and carbon-neutral source of energy, chemicals, and materials. The degradation of residues from forestry and agriculture into simple sugars that can be fermented to ethanol and butanol has the potential to reduce fossil fuel consumption and dependence. A variety of other applications exist in industries such as food processing and feed industry.^3^ It becomes obvious therefore that industrial cellulose degradation has the potential to lead toward a future of reduced greenhouse gas emissions, climate change, and mitigated health impacts from the use of traditional petrochemicals.^4^ Moreover, reduced biodiversity loss and improved animal feeds and human nutrition are also expected outcomes. However, cellulose is highly recalcitrant in nature, and that makes it a challenge to hydrolyze it efficiently.^5^

Since cellulose is such a prevalent source of carbon and energy, various microorganisms have evolved arsenals of biomass-degrading and modifying enzymes to hydrolyze it.^6^ In particular, the filamentous fungus *Trichoderma reesei* is a well-studied organism for the production and characterization of various such enzymes, namely, cellobiohydrolases (CBHs) and endoglucanases (EGs) that are collectively known as cellulases^7^ and break down cellulose. *T. reesei* cellulases are functionally and structurally diverse enzymes.^8^ CBHs or EGs, such as Cel7A, mainly hydrolyze cellulose fibrils from the reducing end and produce cellobiose. Endoglucanases such as Cel7B or Cel5A hydrolyze cellulose internally and hence produce both insoluble cellulose fragments and soluble cellooligosaccharides.^9^ However, cellulases exhibit strong synergistic effects that greatly enhance their efficiency in breaking down cellulose into its constituent sugars, and this is essential for the efficacy of technical cocktails used for saccharification of biomass.^10^ In addition, *T. reesei* cellulases have been engineered to improve their activity and specificity, which can further enhance their hydrolytic efficiency.^11,12^ However, molecular mechanisms that underlie the synergy of cellulases are poorly understood, even to this day.

Cellulase synergy is usually defined quantitatively as the degree of synergy (DS) when the combination of two or more cellulases, of the same or different classes, leads to a higher degree of cellulose chain cleavage than the sum of cleavages of the individual enzymes.^13^ DS is the ratio of the activity of a synergistic mixture to the sum of the activities of the individual components. The DS is typically in the range of 1.5–3, while in some extreme cases, values over 10 has been observed.^14^ One of the oldest hypotheses on endo–exo synergy was proposed by Wood et al.^15^ and states that close proximity between the CBH and EG is required for synergistic action to occur. However, the current understanding and the simplest model of cellulase synergy is that endoacting cellulases, such as Cel7B, create new, free cellulose chain ends, which become new attack points for CBHs (or exocellulases), such as Cel7A.^11^ A separate hypothesis is that the exocellulases uncover internal attack points for the endoglucanases as they depolymerize the cellulose chains.^16^ Another possible explanation of synergy between *T. reesei* cellulases is that Cel7B increases the dissociation rate constant *k*_OFF_ by creating exit points for Cel7A molecules that have been stalled on the cellulose surface.^14^ However, it still remains unclear which mechanism is overarching the synergistic relation of *T. reesei* cellulases, and several studies^14,17−24^ are in disagreement with the current understanding of synergy as described above.

Current understanding of enzyme kinetics and synergy relies heavily on bulk experiments in which the activity of a large number of enzymes is monitored. Such methods allowed researchers to gain a deeper understanding of the factors that influence cellulase activity, such as pH, temperature, and substrate concentration.^25−27^ For example, it was established that *T. reesei* cellulases exhibit a maximum activity at pH = 5 and a temperature of 50 °C. However, the kinetic parameters of the enzymes are extracted as averages over the whole population.^28−33^ Therefore, any information about the behavior and contribution of individual enzymes is lost. On the contrary, microscopy techniques such as atomic force microscopy (AFM) and fluorescence microscopy can provide single molecule data to study the behavior and activity of individual molecules in real-time. In the case of cellulases, single-molecule data provide a unique opportunity to investigate the behavior of these enzymes at the molecular level and to visualize the dynamic interactions between individual enzymes and cellulose fibers. Furthermore, single-molecule imaging has enabled monitoring of the entire reaction cycle: binding, processive motion, and dissociation of CBH Cel7A^34−39^ from the cellulose chains. In particular, total internal reflection fluorescence (TIRF) microscopy has been used to study the behavior of fluorescently labeled Cel7A on the cellulose strands using nonspecific labeling with organic dyes^37^ and quantum dots.^38^ These works provided molecular-level proof that the specific binding of Cel7A molecules to the cellulose is facilitated by the carbohydrate-binding module (CBM) of the enzyme, while CBM-less mutants of the enzyme do not display such specific binding.^37^ Moreover, Haviland et al. measured the speed of the processive motion of Cel7A molecules while they were degrading cellulose chains.^38^ They found that Cel7A typically moved at (3.24 ± 2.68) nm/s for (20.3 ± 38.9) s once it had engaged on the processive hydrolysis of a cellulose segment while also exhibiting static segments before and after a processive run. Specifically, they report the presence of purely static enzymes with a binding duration of 89 s as well as static segments before and after a processive segment with binding durations similar to that of the purely static enzymes. Furthermore, Mudinoor et al.^37^ reported that wild-type (WT) Cel7A exhibited two types of binding, a short-lived one (<15 s) and an essentially immobilized one. Igarashi et al.,^39^ using high-speed AFM measurements, reported the speed of Cel7A to be (3.5 ± 1.1) nm/s. These rates of processive movement measured by single-molecule methods match the results (2–5 nm/s) derived from modeling presteady-state kinetics of bulk systems.^40,41^ In contrast, Jung et al.^34^ report the binding duration of Cel7A of approximately 53 s and rapid displacements with a speed as high as 350 nm/s. These results show great variation but are only focused on one type of enzyme, namely, the CBH Cel7A and therefore provide no single molecule data on the synergy between different classes of cellulases. To address this, Zajki-Zechmeister et al. used high-speed AFM to show that clusters of enzymes are formed in mixtures of exo- and endoglucanases as they synergistically act on the cellulose surface^42^ and that the exoglucanases’ processive cycle accelerates about 100 times compared to that when acting alone.

Here, we used variants of *T. reesei* cellulases engineered with a free cysteine group to specifically label them with a single organic fluorophore. We studied the synergistic behavior between CBHs and EGs in enzymatic cocktails in a wide range of concentrations from the pM range up to 100 nM, while keeping the ratio of exo-to endoglucanase at 10:1.^43^ Using organic fluorophores instead of quantum dots^14^ may circumvent the potential impairment of the enzyme dynamics linked to using labels comparable in size with the enzymes. However, it limited the localization precision of our measurements and made the study of the Cel7A motion during the catalytic step difficult. Still, the experiment is perfectly suitable for measuring how the presence of the synergy partner Cel7B affects the binding kinetics of TrCel7A through its association and dissociation rate constants—*k*_ON_ and *k*_OFF_. Hence, we used single spot detection to measure the residence time of Cel7A on the cellulose chain, both when it was acting alone and in the presence of the synergy partner Cel7B. Through direct observations of Cel7A binding and unbinding on the cellulose, we showed that the addition of Cel7B did not alter the *k*_OFF_ of Cel7A, at either low or high total enzyme concentrations. Interestingly, through measurements of the number of binding events, we found that the arrival rate of Cel7A molecules, the rate at which new molecules bound on the fibrils, decreased with the addition of WT Cel7B in a wide range of enzymatic loads. This could be the direct result of CBH cluster formation in synergistic mixtures as shown previously by high-speed AFM.^42^

## Methods

### Enzyme Variant Design, Expression, and Purification

The enzymes Cel7A and Cel7B are the main CBH and EG from *T. reesei* (Genbank ID CAH10320.1 and AAA34212.1, respectively). Single-site mutations were designed so that a free cysteine residue was introduced on the protein surface to allow specific fluorescent labeling (see the section Enzyme labeling). The resulting mutations were T350C for Cel7A and A303C for Cel7B, following amino acid sequence numbering of the fully mature protein product (residues 18–514 for Cel7A and 23–459 for Cel7B). The TrCel7A variants were recombinantly expressed in *Aspergillus oryzae* and purified using column chromatography procedures previously established.^44,45^ Enzyme purity was confirmed by sodium dodecyl-sulfate polyacrylamide gel electrophoresis (SDS-PAGE) using a NuPAGE 4–12% Bis–Tris gel (Invitrogen, CA, USA) with the protein purity estimated at 98% (Figure S5). Enzyme concentrations were estimated using the theoretical extinction coefficient of 86,760 M^–1^ cm^–1^ for Cel7A.

### Enzyme Labeling

A 10× molar excess of tris(2-carboxyethyl)phosphine (TCEP)was added to the protein, and thiol reduction was allowed to proceed for 30 min in an Eppendorf Thermomixer at room temperature with gentle shaking. The reduced protein was buffer-exchanged to the labeling buffer (50 mM phosphate, 150 mM NaCl, pH 6.5) using a Vivaspin (Sartorius) 20 column with 5 kDa cutoff. The buffer-exchanged protein concentration was measured using a Nanodrop. The selected fluorophore was dissolved in dimethylformamide (DMF). The concentration of the dye stock was measured using absorption spectroscopy. A 10× molar excess of dye was added to the reduced protein, and the reaction was allowed to proceed overnight in a thermomixer at room temperature and 300 rpm. The next day, the reaction mixture was centrifuged in a microcentrifuge at a maximal speed and 4 °C for 20 min to remove any precipitated protein and dye. This was followed by filtering the supernatant through a 0.22 μm syringe filter to remove any residual solids.

### Purification of Labeled Enzyme on Cellulose

Inspired by Moran-Mirabal et al.,^46^ we used microcrystalline cellulose (Avicel PH-101, Sigma-Aldrich, St. Louis, MO) to purify the labeled enzyme. On the day of labeling or the day before, 1.2 g of Avicel was centrifuge-washed three times with 10 mL of deionized water at 1000*g* for 1 min and then three times with labeling buffer. After each solvent exchange, the cellulose was thoroughly resuspended by vortexing. The supernatant was completely removed after the last wash, and the cellulose was stored at 4 °C. Importantly, the amount of cellulose was chosen so that there was a great excess of binding sites for the enzyme to attach, based on the measured density of binding sites on Avicel for Cel7A (0.38 μmol/g, determined by binding isotherm, data not shown).

The labeled enzyme was added to the washed cellulose and mixed. The volume was brought to 5–6 mL with labeling buffer if the mixing was difficult. The tube was sealed with parafilm and covered with aluminum foil and then incubated for 1 h in a thermomixer at 1100 rpm at room temperature. The free dye was removed by centrifuge-washing the cellulose at least 7 times with 10 mL of cold labeling buffer at 1000*g* for 1 min. The supernatant was completely removed after the last wash. The labeled enzyme was eluted three times by adding 5 mL of ethylene glycol, resuspending by vortexing for 15 min, and pelleting the cellulose by centrifugation at 5000*g* for 1 min. The supernatants containing the eluted enzyme were collected and merged. ∼15 mL of eluted enzyme was diluted to 50 mL with cold storage buffer (25 mM (2-(N-morpholino)ethanesulfonic acid) (MES), 100 mM NaCl, pH = 6.0). The enzyme was concentrated using a Vivaspin 20 (5 kDa membrane) at 5000*g* and 25 °C. Any remaining Avicel was removed by filtering through a 0.45 μm syringe filter. Finally, the buffer was exchanged several times with a Vivaspin 20 using cold storage buffer under the same conditions as before to a total dilution factor of 1000.

After purification, the degree of labeling (DOL) was determined by measuring the protein and dye absorbances at 280 and 646 nm, respectively. The DOL was calculated with the equation
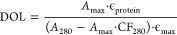
1where CF is a correction factor specific for each dye.

With this procedure, we obtained Cy5-labeled Cel7A-T350C with a DOL of 28%, which was used for all TIRF imaging experiments.

### Activity Assays

The activity assays were performed using 100 nM of enzyme against 70–90 g/L of Avicel in 50 mM sodium acetate buffer with pH 5 at 25 °C according to a well-established procedure described elsewhere.^44,47^ Using this method, we demonstrated the integrity of variant Cel7A-T350C in relation to the WT and after the labeling procedure with Cy5. Activity measurements of the labeled variant, as well as the nonlabeled and WT are shown in Figure S2.

### Glass Surface and Bacterial Cellulose Preparation

Eight-well plates (Ibidi glass-bottomed μ-Slide, catalog number: 80,827) were used in our experiments. Open wells were preferred to sealed coverslips because they provide the possibility of adding and/or removing the solution and to start imaging a short time after the cellulases are added to the cellulose substrate. The wells were thoroughly cleaned to remove dust particles and various other impurities that can induce background noise during single-molecule imaging. We used sonication in Triton-X100 and KOH.^48^ The well plate was subsequently sonicated in 1% Triton-X100 solution, Milli-Q water, 0.1 M KOH, and Milli-Q water for 15 min using separate glassware with Milli-Q wash in between. It was then dried under a nitrogen flow. Bacterial microcrystalline cellulose (BMCC)^49^ was prepared from HCl treatment of bacterial cellulose, using a protocol described previously.^50^ The cellulose was sonicated for 10 min in order to break apart any thick clumps of fibers that would otherwise interfere with TIRF imaging. Five μL of 1 g/L BMCC was immediately deposited on a clean, hydrophilic 8-well plate and was left overnight to fully dry. The next day, 300 μL of green fluorescent beads (Fisher Scientific, 140 nm) acting as fiducial markers, diluted 1000 times from stock, were deposited in each well and left to incubate for 30 min before the solution was removed, and the wells were washed two times with 50 mM sodium acetate buffer, pH = 5. The surface was then incubated with bovine serum albumin at 100 μg/mL for 30 min to prevent nonspecific binding to the glass surface. The solution was then removed, and the wells were again washed twice with the sodium acetate buffer.

### Imaging Buffer

The imaging buffer was based on a standard oxygen scavenging enzymatic system, glucose oxidase and catalase, combined with Trolox.^51^ Trolox was prepared by diluting the powder (Sigma-Aldrich, product number: H0726) in pure ethanol to a 10 mM concentration. Before use, the solution was irradiated with UV for 1 h (Stratalinker, equipped with 312 nm light bulbs). We prepared a 50% w/v glucose stock solution in Tris buffer (pH = 8). Glucose oxidase (Sigma-Aldrich, product number: G2133) was dissolved from powder in 1 mL of 50 mM sodium acetate pH = 5 at 909 nM, and catalase was diluted to 8 μM also in 50 mM sodium acetate (pH = 5). Glucose oxidase and catalase (Sigma-Aldrich, product number: C100) stock solutions were kept at −20 °C and centrifuged at 10,000 rpm for 1 min prior to use. The 50 mM sodium acetate was filtered using 0.2 μm syringe filters before degassing.

The imaging buffer contained 1 mM Trolox, 55 mM glucose, 10 nM glucose oxidase, 0.15 μM catalase in sodium acetate 50 mM, pH = 5. The enzymatic oxygen scavenging system was kept at a minimum concentration to reduce the number of spurious events in TIRF imaging. In order to extend the fluorophore lifetime, sodium acetate was then degassed before use, and a humidified nitrogen flow was kept over the 8-well plate to prevent O_2_ from rediffusing into the solution (see the Supporting Information). The cellulases were diluted on the day of the experiment from stock aliquots (Cel7A-Cy5 3.64 μM, Cel7A-WT 128 μM, and Cel7B-WT 25.1 μM) to the desired concentrations and were kept in the fridge in 2 mL “Protein LoBind” tubes (Eppendorf AG, Hamburg, Germany)^52^ until use. Enzyme solutions after thorough mixing through pipetting were mixed directly in the imaging buffer before the solution was deposited in the well plate.

### TIRF Microscopy

Imaging was done using an inverted Nikon TiU microscope, modified with a homemade objective-based TIRF module^53^ and an electron-multiplying charge-coupled device camera (Evolve 512, Photometrics), a quad-band filter cube (AHF, TIRF quad HC Ex. 390/482/532/640, Em. 446/523/600/677) and a 100×/1.45 oil objective. An intermediate magnification lens was used, bringing the total magnification to 150×, so the pixel size is 107 nm (see the Supporting Information). The TrCel7A-Cy5 conjugate was imaged with the 638 nm laser at 0.36 mW, giving an estimated power density of 12 W/cm^2^, and 200 ms exposure. The electron-multiplying (EM) gain of the camera was calibrated to 165 counts per photons for an EM gain of 500 and a camera gain of 3.^54^ Green fluorescent fiducials were used for autofocusing in MicroManager^55^ every 10 frames. The imaging conditions used for the beads are the 473 nm laser line, 2 mW power, 20 ms exposure, and no gain. The frame rate was 0.5 frames per second. Before the start of the experiment, the optimal TIR angle was identified by moving the motorized mirror and pinpointing the value at which the background signal from the cellulose autofluorescence was minimized. If the imaging position changed during the experiments, the TIR angle was reevaluated to ensure that it had not changed. All experiments were performed at 20 °C.

### Experimental Procedure

For each experimental condition, we recorded three consecutive time-lapse movies of 1000 frames (40 min) each at different positions in the well. So, the first time-lapse started at *t*_0_ = 0 min, the second started 40 min later (*t*_1_ = *t*_0_ + 40 min), and the third started 80 min after the addition of the cellulases to the surface (*t*_2_ = *t*_0_ + 80 min). Note that a different field-of-view (FOV) was used for each time-lapse to probe different areas of the cellulose fibers. The next cellulase solution, usually measured on the same day, was deposited on a fresh cellulose surface prepared in an adjacent well of the plate. To investigate synergy, we added unlabeled Cel7B to labeled Cel7A. In spiking experiments, we also added unlabeled WT Cel7A to increase the total enzymatic load to 10 and 100 nM while keeping the concentration of labeled, i.e., visible Cel7A, in the sub-nM range.

### Processing of Data

The amount of cellulose fibers was estimated using skeletonization of bright-field images in Fiji^56^ after comparison with fluorescence images obtained by staining the cellulose fibers in separate experiments. Tracking of the individual Cel7A molecules in TIRF time-lapse movies was performed in Fiji using the TrackMate plugin.^57^ Through careful filtering and well-defined tracking parameters, we extracted single-molecule tracks for each spot in the raw images (see the Supporting Information). We then extracted the time at which a spot appears and how long it is visible, i.e., the lifetime of the spot. For each frame, we calculated the rate of binding by dividing the number of tracks starting at this frame (*N*) by the duration of a frame (2 s). For a single time-lapse movie, the lifetime histogram was fitted with a double exponential decay. Error on the characteristic lifetime was obtained from the covariance matrix of the fit. We then calculated the residence time and the corresponding *k*_OFF_, taking into account the bleaching of the fluorophore (see the Supporting Information). The error on the lifetime and the bleaching time was propagated and led to the error reported on the *k*_OFF_ values of individual time-lapse movies (see the Supporting Information).

### Single-Molecule Binding Isotherm

In order to generate a binding isotherm from single-molecule data, we kept the number of spots in the image so low that individual binding events could be measured. So, we prepared a dilution series of unlabeled Cel7A from 10 μM to ∼1 pM. We spiked each solution with a fixed volume of Cel7A-Cy5 solution obtained from a dilution series starting at 20 pM and doubling concentration when the number of spots in the field of view became too low. We recorded a time-lapse movie at the same location on the cellulose substrate to ensure that the density of binding sites Γ_max_ is constant. We extracted the number of spots in each frame and calculated the average number of spots in a frame over the whole movie. The number was then scaled with the spiking ratio to calculate the density of bound enzymes Γ by dividing with the area of our FOV since it was fully covered by cellulose. As done by Peterson et al.,^58^ who studied the kinetics of DNA hybridization, we plotted the spot density Γ, scaled using the spiking ratio, and fitted using . This allowed us to estimate the Γ_max_ and *K*_a_.

## Results

**Figure 1:**
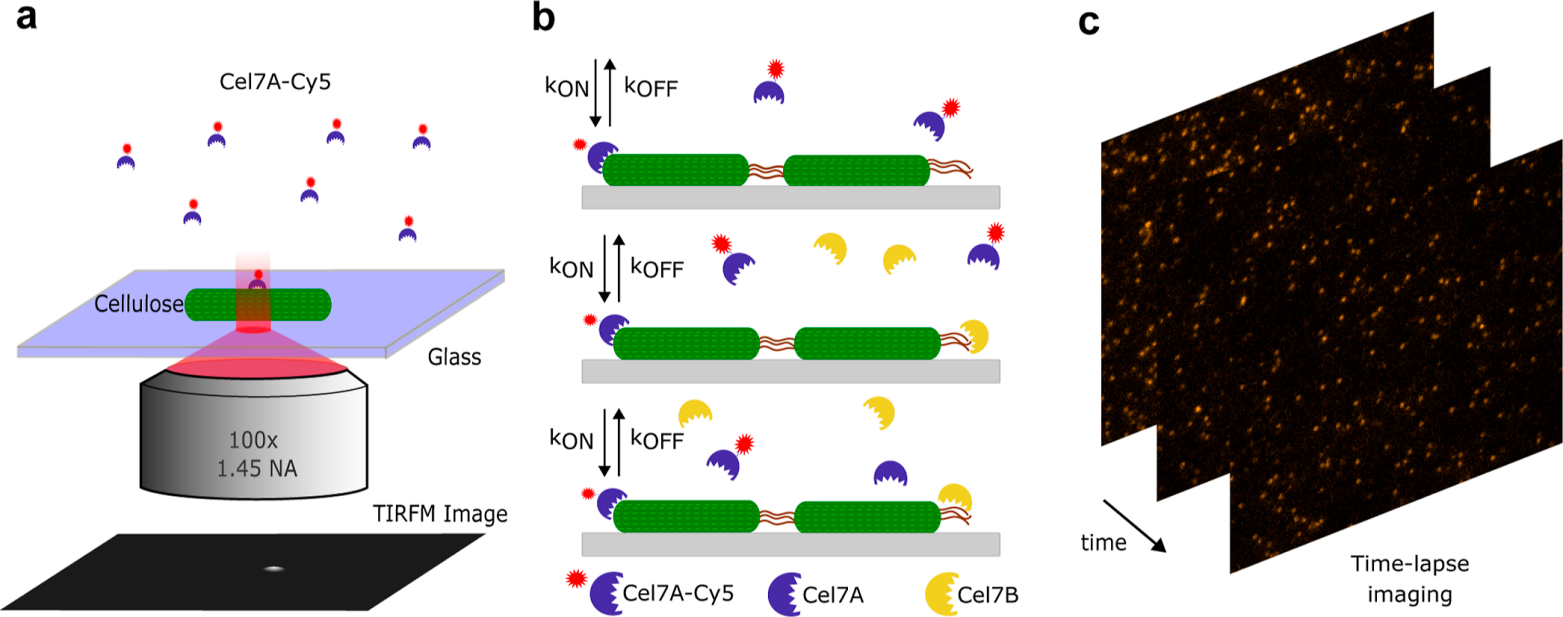
Single-molecule microscopy of *T. reesei* Cel7A: (a) schematic illustration of the objective-based TIRF setup used to image the binding of Cel7A labeled with Cy5 to bacterial cellulose fibrils. (b) We compared three enzymatic compositions: labeled Cel7A alone, a synergetic mix composed of labeled Cel7A and unlabeled Cel7B in a ratio of 10:1 and the same synergetic mix where the enzymatic load is adjusted with unlabeled Cel7A. (c) Time-lapse TIRF imaging (56 × 56 μm^2^ FOV) of labeled Cel7A in solution acting on cellulose immobilized on a glass surface.

We used fluorescence time-lapse imaging to measure the binding kinetics of Cel7A under conditions where enzyme synergy occurs. So, with TIRF microscopy, we imaged Cy5-labeled Cel7A (Figure 1a) binding to bacterial cellulose fibrils immobilized on a glass surface either alone or in a 10:1 mixture with nonlabeled Cel7B (Figure 1b).

### Mutant Enzyme Engineering and Labeling

Through site-directed mutagenesis of *T. reesei* cellulases, we designed and introduced free cysteine residues on CBH Cel7A. In this study, we focused on a Cel7A mutant, which had a threonine at position 350 substituted with a cysteine. For a comprehensive analysis of our exploration of other positions, refer to Figure S6 and Table S1. The mutants exhibited kinetic parameters similar to those of their respective WT enzymes, indicating that they remained functional (Figure S1).

The cysteine modification allowed us to specifically label the enzymes with a single organic fluorophore, which limited the risk of loss of activity. One of the native cysteines in the enzyme core and one in the CBM were exposed to the solvent (see Figure S7), so, in principle, the sample of mutant Cel7A used in our experiments contained enzymes labeled on a native cysteine, the mutant cysteine, or both. However, the formation of the disulfide bridges by the cysteine residues is known to be critical for the enzyme’s activity.^59−62^ Therefore, the labeling of the native cysteines was expected to lead to Cel7A with impaired functionality so that Cel7A interacts weakly with the cellulose. In this work, the purification protocol based on cellulose ensured that any enzymes that were made inactive following native cysteine labeling were removed. The increased activity of the labeled mutant (116% of the nonlabeled) suggested that this indeed occurred. From these observations, we concluded that any enzymes made inactive due to labeling of native cysteines were likely removed in this process. Moreover, we chose fluorophores that are hydrophilic to avoid interaction with the purification column. We thus used maleimide sulfo-Cy5^37^ and Alexa Fluor 568. For both fluorophores, purification by size exclusion chromatography resulted in very poor activity compared to the activity that the enzyme exhibited before the labeling. We performed a labeling experiment where WT Cel7A was labeled with Sulfo-Cy5. The low purification yield (3–8%) of that experiment suggested that the enzymes were strongly bound to the purification column and did not elute or eluted at a later point in time. The column used (HiPrep 26/10) has a cross-linked dextran matrix, which is made up of glucose molecules, much like cellulose. This suggests that enzymes that eluted were weakly interacting with the column, and enzymes that retained their affinity to cellulose were stuck. Moreover, Cel7B was successfully eluted from the purification column, as opposed to Cel7A, because its lower affinity for cellulose^49^ reflects on a lower affinity for the column matrix. Cy5-labeled Cel7A was thus purified from free fluorophore using cellulose as matrix.^46^ With this technique, we were able to achieve 28% DOL for Cel7A-Sulfo-Cy5, and 116% activity of the enzyme was retained compared to that in the nonlabeled enzymes. Our hypothesis for the extra 16% was that functional enzymes were concentrated during cellulose purification, resulting in increased activity.

### Cel7A Residence Time and Binding Rate Analysis

**Figure 2:**
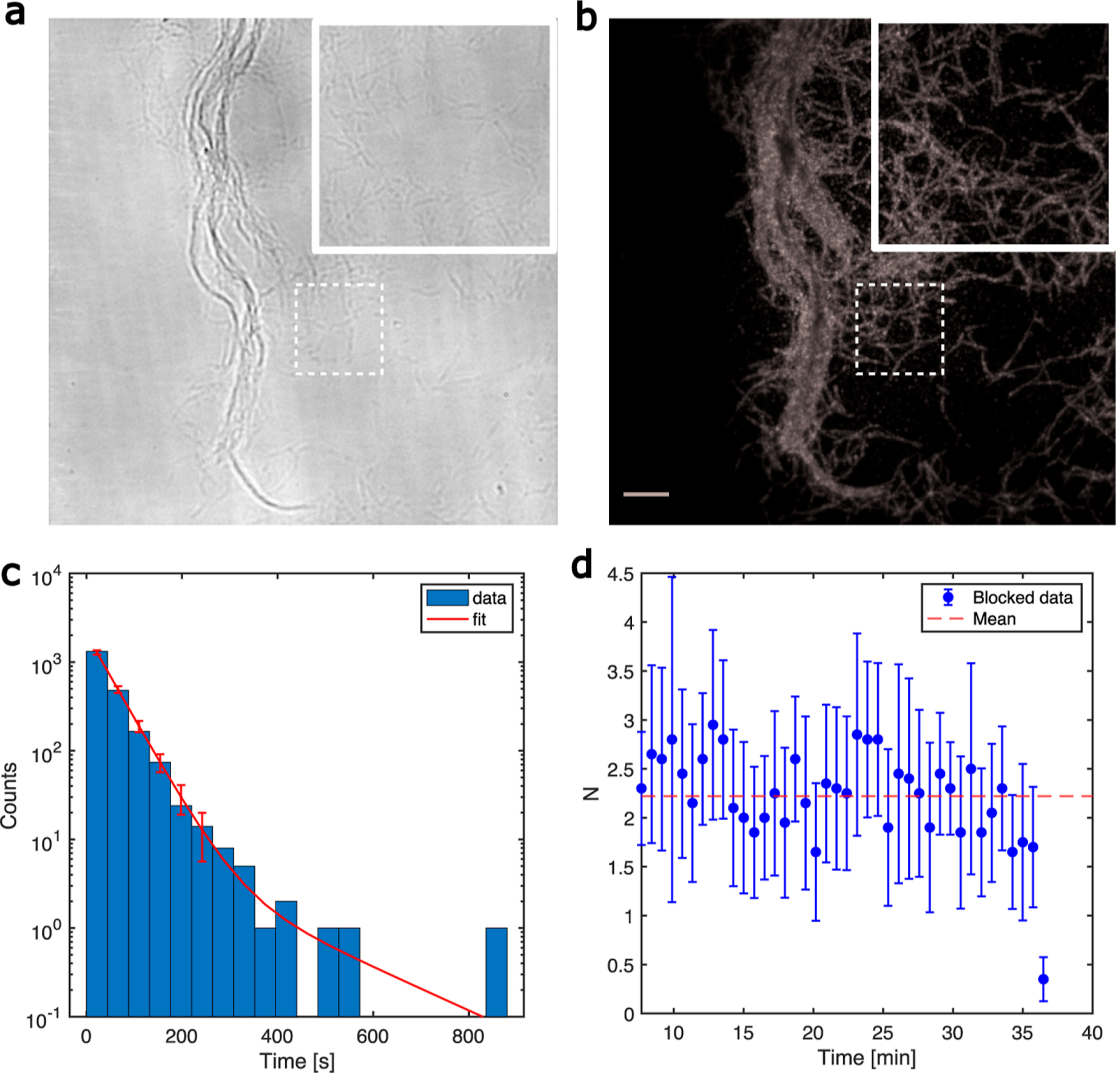
Residence time and binding rate analysis of *T. reesei* Cel7A: (a) bright-field image of individual and aggregated cellulose fibrils immobilized on the glass surface. (b) Maximum intensity projection of a 1000-frame TIRF time-lapse sequence depicting 1 nM Cy5-labeled Cel7A molecules attached to those cellulose fibrils. Binding of the enzyme is specific as indicated by the lack of enzymes bound on the glass surface. (c) Histogram of the spot duration as acquired from the time-lapse stacks. A double exponential decay fit is shown in red. Error bars are the  of the expected number of counts on each bin. (d) Mean number of new binding events per frame blocked per 20 frames (<1 min) for one time-lapse movie. Error bars are the standard error of the mean (sem).

Using the Cel7A-T350C variant labeled with Cy5, we acquired three 1000-frame time-lapse movies at different positions on the cellulose substrate (Figure 1c) for each enzyme mixture tested. Cel7A-Cy5 molecules bound very specifically to the cellulose fibrils, as indicated by the lack of enzymes bound to the glass surface (Figure 2a,b). For every movie acquired, we detected single-molecule binding and plotted the duration of binding events (see the Supporting Information). The binding events with duration less than 10 s were discarded as they were considered to be either short-lived CBM-mediated association to the cellulose or enzymes in solution diffusing through the TIR evanescent field without binding to the substrate.^38^ We also discarded spots that were present at the first frame or the last frame of the imaging sequence since in those cases, determining the duration of the binding event was not possible.

The resulting binding duration histogram consistently showed a double exponential (Figure 2c) as expected from previous bleaching time experiments with Cy5,^37^ and it was also evident in our own characterization of Cy5 at our imaging conditions (Figure S10).

We fitted the data with models that were the sums of exponentials. For each model, we did chi-squared goodness-of-fit tests (significance level α = 0.05). The tests showed that a single-exponential model was not consistent with the data (*p* < 0.05), while a double-exponential was (*p* = 0.2–0.8). So, the double-exponential model was the exponential model with the fewest parameters that were consistent with the data. For this model, the fit gave the two characteristic time scales that were 46 s, corresponding to short-lived spots, and approximately 170 s for the long-lived spots (Figure 2c).

The number of events contributing to the tail of the histogram, described by the second exponential of the model, was low. So, the relative error in the fitting parameter was large. In contrast, the first exponential decay with a characteristic time of τ_meas_ = 46 s was a robust measure of the residence time of Cel7A molecules that bound tightly to the cellulose and which has been shown to dominate in the concentration range we operated in (below 10 nM).^63^ Here, we note that since the characteristic bleaching time of the fluorophore (τ_bleach_) and the spot duration (τ_meas_) were on the same order of magnitude, we need to take bleaching into account when calculating the residence time of the enzyme and the unbinding rate *k*_OFF_ using spot duration τ_meas_. Briefly, the spot duration is the minimum time of two events: the dissociation of the enzyme and the bleaching of the fluorophore. We get that 1/τ_res_ = 1/τ_meas_ – 1/τ_bleach_ with τ_bleach_ being the characteristic bleaching time of Cy5 and τ_res_ being the residence time of the enzyme on the cellulose fibrils. So, the measured spot duration of 46 s corresponded to a residence time of 79 s once corrected for the bleaching time of 110 s (see the Supporting Information). In our experiments, we used a laser power of 0.4 mW that minimizes bleaching while still ensuring a sufficient single-fluorophore signal over the background. Lower powers would compromise the reliability of single-molecule localization. To address the issue of fluorophore photobleaching at this power, we implemented a robust mathematical correction rather than reducing the illumination power further. Also, we plotted the number of every new binding event in each frame versus time to extract the binding rate of the enzyme *r*_ON_ (Figure 2d).

**Figure 3:**
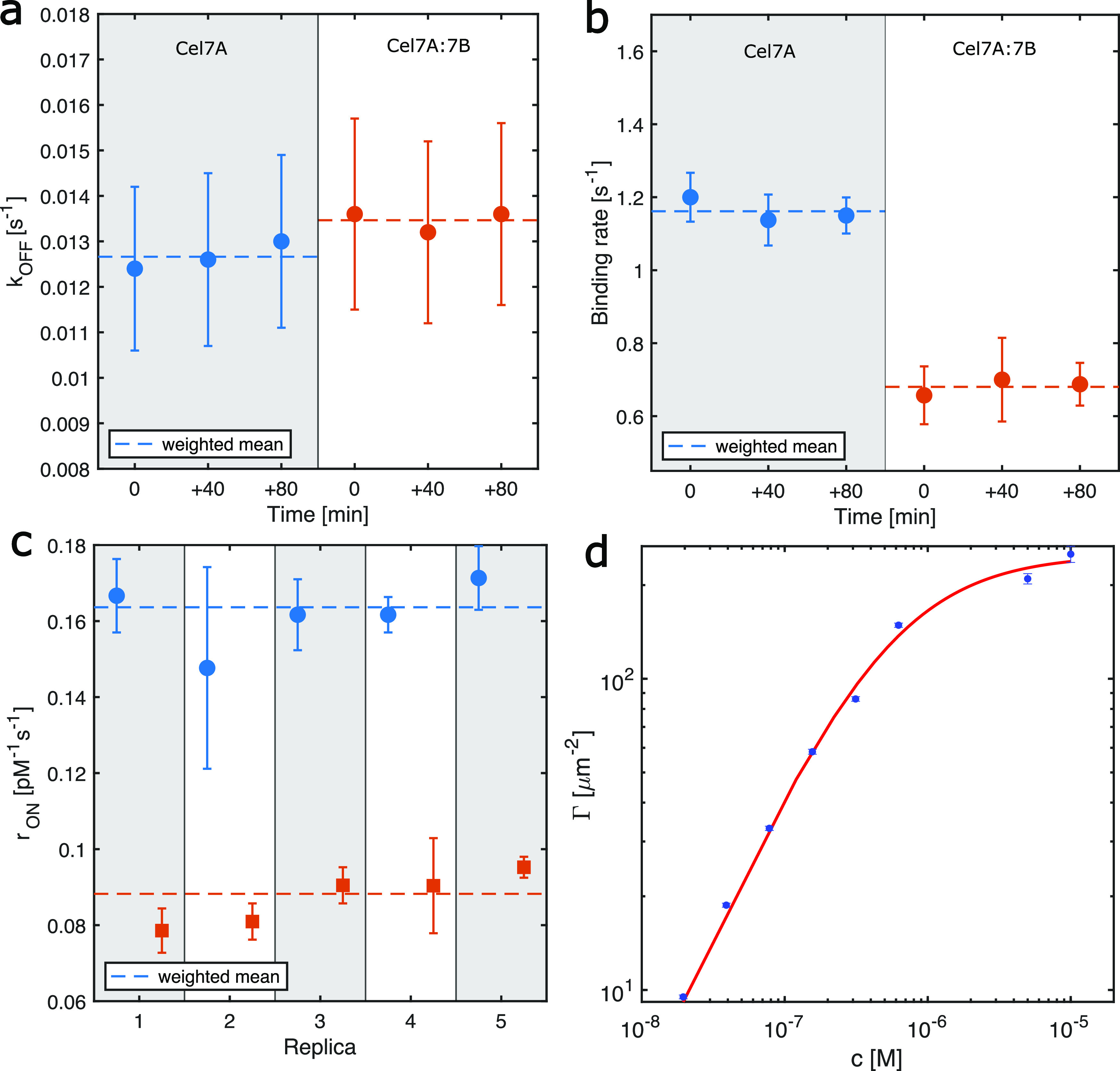
Kinetics of *T. reesei* Cel7A binding, alone and in a synergistic mixture. (a) *k*_OFF_ for the nonspiked labeled Cel7A experiments, calculated as the inverse of the residence time. Cy5-labeled Cel7A when it is acting alone or in the presence of Cel7B. Error bars are propagated from the error on fitting parameters of individual residence time histograms. (b) Rate of binding when Cel7A is alone and in the presence of the synergy partner Cel7B. Error bars are propagated through the s.e.m of each time-lapse movies. (c) Molar binding rate constant *r*_ON_ for paired experiments where measurements for Cel7A alone (circles) and measurements in the presence of Cel7B in the ratio 10:1 (squares) are compared on the same day. Error bars are calculated through error propagation over three time-lapse movies. The dashed lines represent the weighted means. (*N* = 5). (d) Single-molecule isotherm of Cel7A alone with fit to obtain Γ_max_ is (250 ± 8) μm^–2^, and *K*_a_ is (1.9 ± 0.2) × 10^6^ M^–1^.

We first measured the binding kinetics of Cel7A-Cy5 when alone and in the presence of synergy partners at concentrations so low that only isolated single molecule spots occurred on the cellulose surface. Our data showed that in such experiments (Figure 3a), the Cel7A dissociation rate constant *k*_OFF_ = 1/τ_res_, calculated as the inverse of the residence time, remained unchanged over the course of the experiment, adding up to 120 min *k*_OFF_^7A^ = (0.013 ± 0.002) s^–1^. Most importantly, however, the residence time of Cel7A did not appear to be significantly affected by the addition of Cel7B that acts synergistically with Cel7A *k*_OFF_^7A:7B^ = (0.013 ± 0.002) s^–1^ as the values are consistent with a common mean of 0.013 s^–1^. Therefore, we concluded that the dissociation rate constant *k*_OFF_ of the CBH did not increase with the addition of the synergy partner Cel7B. The values of *k*_OFF_ obtained here are well within the range for the rate constants for dissociation (0.0007–0.14 s^–1^) of the cellulose chain from the Cel7A active site,^64^ and they are summarized in Table 1.

**Table 1:** Summary of *k*_OFF_ and *r*_ON_ Values with No Unlabeled Cel7A Added^a^

variable	experimental condition	
	nonspiked	
	Cel7A	Cel7A:Cel7B
*k*_OFF_ (s^–1^)	0.013 ± 0.002	0.013 ± 0.002
*r*_ON_ (pM^–1^ s^–1^)	0.16 ± 0.03	0.08 ± 0.01

a Values are averages over all experiments performed for each concentration. Error bars are calculated through error propagation.

In our data, we also quantified the rate at which new spots appear. The rate referred to as the binding rate corresponds to the rate at which Cel7A-Cy5 molecules bind to the cellulose fibrils during the steady state. We showed that the binding rate of Cel7A molecules remained the same throughout the 120 min of acquisition when it acted alone (Figure 3b) and was consistent with a mean of (1.1 ± 0.1) s^–1^, indicating that the binding rate of Cel7A molecules did not change during the course of our experiment. We note that the molar binding rate constant *r*_ON_ values obtained here (Figure 3c) cannot be directly compared to literature values obtained through bulk assays as they include the number of binding sites probed. We thus generated a single-molecule isotherm to extract the density of binding sites Γ_max_ and the molar association rate constant *k*_ON_ of the Cel7A-Cy5 in our experiment. We spiked unlabeled Cel7A with the labeled Cel7A-Cy5 at a known spiking ratio. From the isotherm fit (see the Methods section), we got Γ_max_ and *K*_a_ = *k*_ON_/*k*_OFF_ and found that Γ_max_ = (250 ± 8) μm^–2^ and *K*_a_ = (1.9 ± 0.2) × 10^6^ M^–1^. This corresponded to *k*_ON_ = (8.8 ± 1.5) × 10^4^ M^–1^ s^–1^ using the *k*_OFF_ for Cel7A we determined earlier and scaling with the degree of labeling of 28%. The measured molar rate constant *k*_ON_ can be converted to an association rate using the enzyme concentration. Cruys-Bagger et al.^40^ used *c* = 1.5 × 10^–7^ M cellulases, and at this concentration, our *k*_ON_ = (8.8 ± 1.5) × 10^4^ M^–1^ s^–1^ corresponds to an association rate of 10^–2^ s^–1^. At this concentration, Cruys-Bagger et al.^40^ report a molar rate constant of 10^–2^ (g/L)^−1^ s^–1^. With a cellulose concentration of 3 g/L, their reported *k*_ON_ corresponds to an association rate of 3 × 10^–2^ s^–1^, in good agreement with our findings. Furthermore, we can express *k*_ON_ in terms of binding site concentration (molar concentration). Assuming full coverage of the well bottom surface with cellulose, the density of binding sites is Γ_max_ = 250 μm^–2^. For the total surface area of 1 cm^2^, we found that *k*_ON_ = 10^7^ M^–1^ s^–1^, where the concentration refers to the binding sites. Cruys-Bagger et al.^33^ reported *k*_ON_ = 0.33 × 10^6^ M^–1^ s^–1^, in reasonable agreement with our results.

We could have generated a single-molecule isotherm for the other experiments; however, as this is cumbersome, we instead made sure to keep the density of cellulose Γ_max_ constant so the measured variations of *r*_ON_ reflect the changes in *k*_ON_.

Our most important finding is that the binding rate of Cel7A in the presence of the synergy partner Cel7B was almost a factor of 2 lower, (0.6 ± 0.1) s^–1^, compared to that when it was acting alone (Figure 3b). Moreover, taking the Cel7A concentration in replica experiments into account, we calculated the molar binding rate constant *r*_ON_ of Cel7A (Figure 3c). Here, *r*_ON_ = *N*·*k*_ON_, where *N* is the amount of binding sites in the FOV, and therefore dimensionless, and *k*_ON_ is the molar association rate constant. We saw that *r*_ON_ values were consistent with an average of (0.16 ± 0.03) pM^–1^ s^–1^ when Cel7A was acting alone, while *r*_ON_ was (0.08 ± 0.01) pM^–1^ s^–1^ in synergistic mixtures, where Cel7B was present. These values are summarized in Table 1.

**Figure 4:**
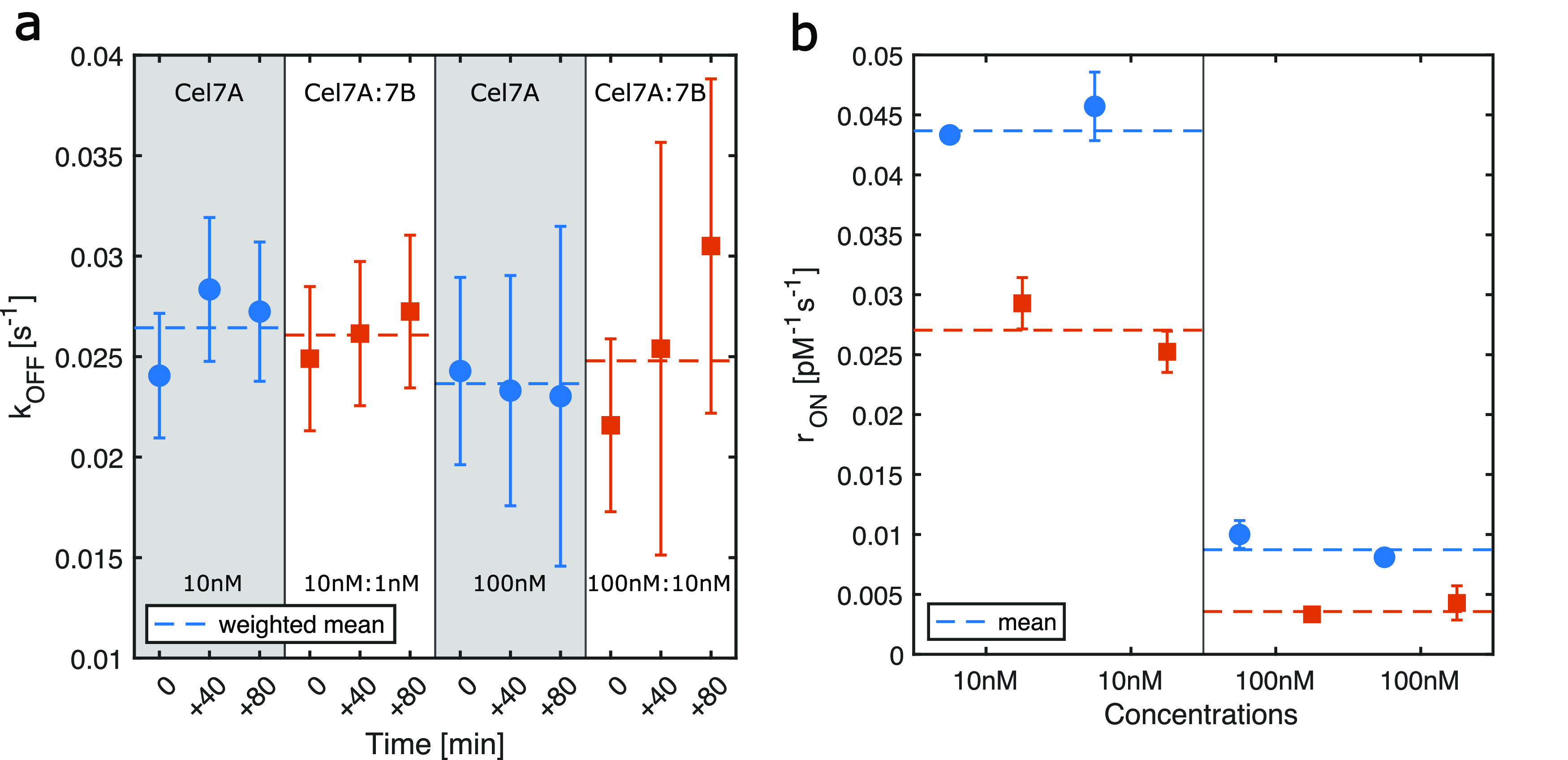
Binding kinetics of *T. reesei* Cel7A at higher total enzyme concentrations. The concentration of enzymes was increased by adding unlabeled enzymes, so the concentration of labeled Cel7A was kept low. (a) *k*_OFF_ at 10 nM WT Cel7A:1 nM WT Cel7B:7 pM Cel7A-Cy5 and at 100 nM WT Cel7A:10 nM WT Cel7B:7 pM Cel7A-Cy5. Error bars are obtained from the covariance matrix of the fit to individual histograms. The weighted mean is shown for each group. (b) Molar binding rate constant *r*_ON_ for spiked experiments for all experiments performed. Error bars were calculated through error propagation of the s.e.m of individual time-lapse movies. The weighted mean is shown as a dashed line for replica experiments (*N* = 2). The blue circles are Cel7A alone, and the red squares are Cel7A:Cel7B mixture.

Next, we probed the kinetics of Cel7A at higher concentrations to simulate the biochemical bulk experiments traditionally performed to study cellulases’ kinetics. We increased the total enzyme concentration by adding WT Cel7A, keeping the concentration of labeled Cel7A in the pM range, so the number of bright spots in the TIRF images was at a single-molecule-suitable density. Similar to the previous experiments, we compared *k*_OFF_ values of labeled Cel7A when acting alone and when in the presence of Cel7B at a total concentration of 10 and 100 nM (Figure 4a). No significant change of *k*_OFF_ appeared, and the total enzyme load (10 and 100 nM) did not seem to affect this either. However, by comparing the acquired *k*_OFF_’s at pM concentrations (Figure 3a) and at higher concentrations (Figure 4a), we saw that increasing the total enzymatic load caused a faster dissociation of Cel7A. Specifically, there was a 100% increase of the *k*_OFF_ in spiked experiments compared to that in the nonspiked experiments. Possibly, competition for binding sites at a high enzymatic load may cause Cel7A molecules to dissociate faster from the cellulose fibrils. Finally, we note that the reduction of *r*_ON_ observed earlier in the nonspiked experiments persisted here as well (Figure 4b). The arrival rate of newly labeled Cel7A molecules was reduced by half when Cel7B was added to Cel7A, indicating that a reduction of *r*_ON_ is specific to the mode of action of Cel7A on the cellulose fibrils in the presence of its synergy partner, endoglucanase Cel7B. The results for the *r*_ON_ and *k*_OFF_ rate constants for our spiked experiments are summarized in Table 2.

**Table 2:** Summary of *k*_OFF_ and *r*_ON_ Values with Unlabeled Cel7A Added^a^

variable	experimental condition			
	spiked			
	10 nM:1 nM:7 pM		100 nM:10 nM:7 pM	
	Cel7A	Cel7A:Cel7B	Cel7A	Cel7A:Cel7B
*k*_OFF_ (s^–1^)	0.026 ± 0.004	0.026 ± 0.004	0.024 ± 0.006	0.026 ± 0.008
*r*_ON_ (pM^–1^ s^–1^)	0.0445 ± 0.0017	0.0273 ± 0.0019	0.0091 ± 0.0009	0.0038 ± 0.0095

a Values are averages over all experiments performed for each concentration. Error bars are calculated through error propagation.

**Figure 5:**
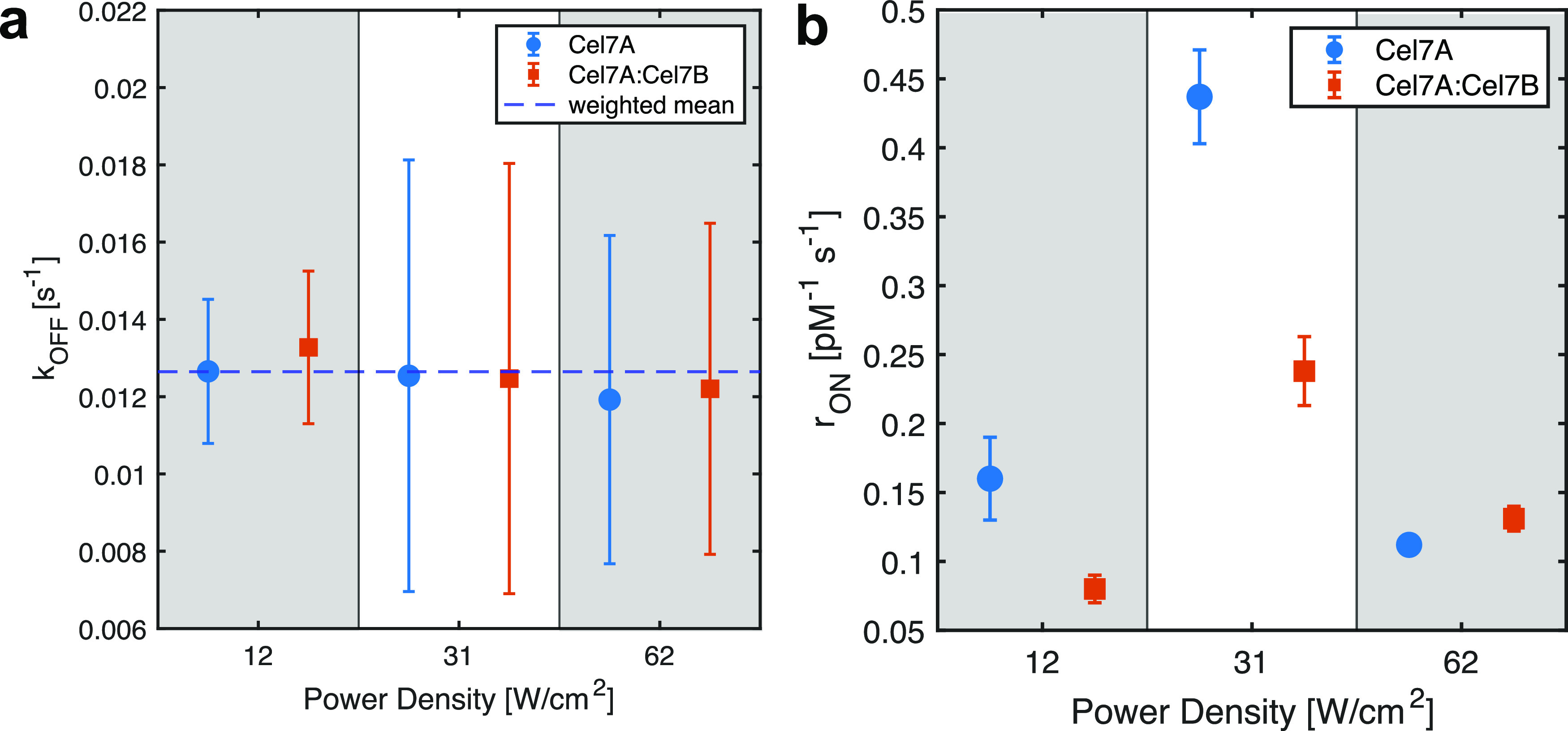
Measurements at increased illumination. The illumination power was increased from 0.4 to 1 and 2 mW corresponding to 12, 31, and 62 W/cm^2^. (a) *k*_OFF_ of Cel7A alone (dots) and in the presence of Cel7B (squares) is constant after correction for the reduced spot lifetime due to photobleaching. (b) Reduction of the molar binding rate constant *r*_ON_ upon addition of Cel7B persists at 31 W/cm^2^ but vanishes at 62 W/cm^2^. Error is obtained as described previously.

Up to this point, we performed measurements at low excitation power to maximize the lifetime of the fluorophore while preserving a good signal-to-noise ratio. Next, we increased the illumination power to detect cellulase cluster formation reported by others.^42^ At higher illumination, the bleaching time of single fluorophores was, on average, comparable to or even shorter than the residence time of the enzyme (Figure S11). However, we were able to calculate the *k*_OFF_ of the enzymes by correcting for fluorophore bleaching (Figure 5a). Despite the increased power and severe photobleaching, we measured a constant *k*_OFF_. This highlights the robustness of the correction that we implemented as well as our analysis of the residence time of the enzyme on the cellulose fibril. The measured arrival rate and the calculated *r*_ON_ are shown in Figure 5b. Although the rate was affected by bleaching, we did not expect the behavior of the enzyme to be affected by the illumination intensity. At intermediate power, we counted more binding events, but this is due to the quality threshold we applied: only spots with intensities significantly higher than the background were detected, which corresponded to a spot quality threshold (see the Methods section). Only enzymes with intensities above the intensity threshold were distinguished from the background and localized by the algorithm. As the enzymes were imaged with TIRF microscopy and the TIR field decays exponentially with distance from the surface, the enzymes situated slightly higher on the cellulose fibrils, (i.e., further away from the surface) lead to less intense spots possibly below the threshold. At higher laser power, those enzymes then led to spots with an intensity that exceeded the threshold. Therefore, they were then counted as binding events and used in the calculation of *r*_ON_. So, the increased *r*_ON_ was not due to enhanced enzyme binding but rather a result of more enzymes passing the detection threshold. Nevertheless, we saw that the reduction of *r*_ON_ upon the addition of Cel7B persisted at an intermediate power (31 W/cm^2^).

However, when bleaching was severe, we counted more spots in synergistic mixtures than for Cel7A alone. At first glance, this may be surprising, but given the threshold on spot duration applied in data processing, we discarded events shorter than 10 s since the majority of those might be due to weak binding or diffusion in solution through the TIR excitation. However, at increased illumination, bleaching was more severe, and the spot duration was significantly reduced. So, we discarded more of the specific Cel7A binding events. Consequently, only spots that persisted longer than the duration threshold were used in the analysis. Notice that spots that pass the threshold had a longer duration than what would be expected for single fluorophores under the same illumination conditions (Figure 5b). The calculated values for the rate constants at increased illumination powers are summarized in Table 3.

**Table 3:** Values for Experimentally Evaluated Variables *k*_OFF_ and *r*_ON_ for the Experiments Performed at Increased Illumination Powers^a^

variable	experimental condition			
	increased illumination power (W/cm^2^)			
	31		62	
	Cel7A	Cel7A:Cel7B	Cel7A	Cel7A:Cel7B
*k*_OFF_ (s^–1^)	0.013 ± 0.006	0.012 ± 0.006	0.012 ± 0.004	0.012 ± 0.004
*r*_ON_ (pM^–1^ s^–1^)	0.437 ± 0.034	0.238 ± 0.025	0.112 ± 0.007	0.131 ± 0.009

a Values are averages over all experiments performed for each concentration. Error bars are calculated through error propagation.

## Discussion

### Cel7A Dissociation Rate Constant *k*_OFF_ Is Unaffected by the Addition of Cel7B

We extracted the characteristic spot duration from a fit of the spot duration distribution (Table 4). However, the duration of a spot resulted from the enzyme residence time and the bleaching time of single fluorophores, so we calculated the residence time of the enzymes using the spot duration and the characteristic bleaching time of the dye Cy5 under our imaging conditions (see the Supporting Information). The correction was non-negligible and lowered the *k*_OFF_ values from 0.020 to 0.013 s^–1^, highlighting the importance of such a correction in single-molecule fluorescence assays.

**Table 4:** Correlation of Observed Binding and Dissociation Rate Constants with Mechanistic Insights for Cellulases

variable	exp. condition	result	mechanistic model
*k*_OFF_	nonspiked and spiked	*k*_OFF_^7A^ = *k*_OFF_^7A:7B^	synergy does not manifest in *k*_OFF_
*r*_ON_	nonspiked and spiked	*r*_ON_^7A^ = 2·*r*_ON_^7A:7B^	cellulase cluster formation
*k*_OFF_	spiked	*k*_OFF_^spiked^ > *k*_OFF_^nonspiked^	CBM-mediated binding concentration effect
*r*_ON_	spiked	*r*_ON_^spiked^ < *r*_ON_^nonspiked^	concentration effect

The enzyme residence time is a combination of the time the enzyme spends hydrolyzing as well as the time it remains bound but is unable to hydrolyze the cellulose fibril. We showed that the *k*_OFF_ of Cel7A remained unchanged (Figure 3) during the course of an experiment (∼120 min), indicating that the catalytic behavior of the enzyme was unchanged as well. Moreover, the unbinding behavior of Cel7A did not seem to be affected by the presence of the endoglucanases Cel7B. One model of endo–exo synergy suggests that Cel7B acts by creating exit points of stalled Cel7A molecules, thereby increasing *k*_OFF_.^14^ However, our results suggested that the endoglucanase did not alter *k*_OFF_ in the synergy cocktails we used (concentrations ranging from pM to μM), which are similar to those in bulk assays.

### CBM- and Catalytic Domain (CD)-Mediated Binding of Cel7A

We now consider the spiked experiments where the total concentration of Cel7A is increased to the 10–100 nM range by the addition of nonlabeled WT Cel7A while keeping the concentration of Cel7A-Cy5 in the pM range. Here, Cel7A exhibited a higher *k*_OFF_^7A^ = (0.025 ± 0.004) s^–1^ compared to that in nonspiked experiments, when acting alone and in the presence of Cel7B *k*_OFF_^7A:7B^ = (0.026 ± 0.006) s^–1^. A possible explanation for this increase is that at such high concentrations of enzyme, the main mode of binding is through CBM-mediated binding. Jalak and Väljamäe have shown that for enzyme concentrations below ∼10 nM (conditions of our nonspiked experiments), the Cel7A molecules are bound almost exclusively through their active site (“strong mode” in Jalak and Väljamäe) so they can be either hydrolyzing the cellulose or prevented from movement by irregularities on the surface.^63^ In the case where the enzyme is bound solely through the CBM and cannot start a catalytic cycle, it is expected to unbind faster than it would if it was bound through the active site. This effectively leads to an increase of the *k*_OFF_.

Comparing our results on the *k*_OFF_ behavior in synergistic mixtures with previous work,^14^ we found that they disagreed with the previously reported increase of *k*_OFF_ in the presence of the endoglucanase. However, the two assays are inherently different: Jalak et al.^14^ utilized much higher concentrations of enzymes of CBHs and EGs (in the μM range) compared to our nonspiked, sub-nM concentrations. Furthermore, the addition of β-glucosidase along with the exo- and endocellulases drives the recruitment of Cel7A by avoiding product inhibition. For our spiked experiments, where the total concentrations of enzymes matched those used by Jalak et al., our results also indicated an increased *k*_OFF_ and therefore a faster recruitment of the CBH. However, this behavior of *k*_OFF_ was apparent also when Cel7A was acting alone on the cellulose fibril. In our experiment, the change of *k*_OFF_ is dominated by concentration effects and not by synergy effects.

### Cel7A Apparent *k*_ON_ Decrease with Addition of Cel7B

#### Cellulase Cluster Formation

Interestingly, our findings suggested a reduced association rate constant in the presence of Cel7B in a cocktail of 10:1 Cel7A:Cel7B. The measured *r*_ON_ of Cel7A was about half of what it was when Cel7A acted alone (Figure 3). We also observed that the measured *r*_ON_ decreased as the concentration of Cel7A increased in our spiking experiments, also when Cel7A was acting alone (Figure 4). This reduction of the apparent binding rate constant of the Cel7A-Cy5 molecules originates from the fact that in spiked experiments, a majority of the bound enzymes were unlabeled. This statement may be qualified by the binding isotherm (Figure 3d) and data from bulk measurements, which showed that half-saturation of cellulose occurred at a Cel7A concentration of approximately 100 nM.^65^

This observation confirmed that in the nonspiked experiments, there was an excess of binding sites. So, the observed reduction of the *r*_ON_ in the presence of Cel7B has another origin. The *r*_ON_ was stable throughout the 120 min of the experiment with Cel7A, and it only decreased when Cel7B was added to the mixture and then remained stable for the next three measurements (120 min). Here, we note that because of the limited lateral optical resolution of the technique, the appearance of a spot on the surface might be due to the simultaneous binding of one or more enzymes within the diffraction limit (∼280 nm). So, the reduction of the measured molar binding rate constant might be compatible with the formation of clusters of enzymes on the cellulose. It was recently shown using AFM that the presence of Cel7B and Cel7A in a mixture leads to the formation of transient clusters of, on average, three enzymes.^42^ In the scenario where enzymes form clusters when both types of enzymes are present, the measured reduction in the frequency of new binding events might be due to the fact that a spot now contains several Cel7A enzymes. This does not imply that the total amount of enzymes adsorbed is diminished. In summary, we saw a reduction in the apparent *k*_ON_, together with the invariance of *k*_OFF_, so our data support the hypothesis of cluster formation as a mechanism of synergy.

We labeled Cel7A with single fluorophores, so clusters could in principle be identified in our data as brighter spots than single Cel7A molecules. However, detecting clusters solely based on an intensity analysis is difficult. First, the histogram of spot intensities was broader than in a classical biophysical assay on surfaces due to the height distribution of the cellulose fibers. As fibers may extend away from the surface, the excitation strength of the TIR evanescent wave was broadened, which prevented us from detecting clusters in a classical spot intensity analysis. A better indication of the presence of clusters is given when working at increased illumination, where bleaching of the fluorophore becomes the limiting factor of the spot duration. Here, we saw that the Cel7A:Cel7B samples yielded more detected spots than that by Cel7A alone. This observation was consistent with the formation of clusters, considering that the spots were only detected in the microscopy images when they were above a minimum intensity and had a certain duration (Figure 5b). In the hypothesis of cluster formation, a spot should contain, on average, more than a single fluorophore. Such spots are expected to exhibit a longer lifetime because the lifetime of the spot always corresponds to the fluorophore bleaching last. In other words, in the presence of clusters, the spot duration is systematically longer than that for single enzymes even if the residence time of the enzyme is the same (Figure 5a). Consequently, cluster formation should lead to more spots being detected, and this is what we observed at higher illumination where the reduction of *r*_ON_ in the presence of Cel7B seemed to vanish.

Conversely, it was proposed that these clusters enable the exocellulase to complete its catalytic cycle much faster than when acting alone and hence lead to synergy.^42^ In this scenario, the reduction in the measured binding rate could be concomitant with an increase in the dissociation rate. This is not what we observed, probably because most of the residence time of Cel7A represents nonhydrolyzing steps of the catalytic cycle, and not the hydrolyzing step itself.

#### Substrate Reactivity Drop Mechanism

An alternative mechanism that could lead to a reduced *k*_ON_ of Cel7A in the presence of Cel7B is the so-called substrate reactivity drop mechanism.^66^ This mechanism states that an enzyme hydrolyzes the most easily accessible substrate first and then proceeds to the more recalcitrant substrate. Consequently, the catalysis slows during the ongoing reaction. The bacterial cellulose used here is highly crystalline and hence recalcitrant.^67,68^ Therefore, Cel7A is expected to act on amorphous cellulose first because it is immediately available and then proceed to the crystalline parts.^66^ However, the endoglucanase Cel7B is faster on amorphous cellulose than on Cel7A,^49,66^ and therefore, it can deplete the easily digestible amorphous substrate faster than Cel7A can act. So, we expect that Cel7B addition induces a depletion of easily accessible attack sites for Cel7A to bind to, leading to a decrease of the Cel7A binding rate. The Cel7A molecules that do bind could do so on newly generated reducing ends of cellulose fibrils by Cel7B.

## Conclusions

In conclusion, we labeled Cel7A with single fluorophores and established a purification protocol that produced variants with the same activity as the WTs. Using this approach, we were able to reliably measure the molar association and dissociation rate constants of Cel7A in synergy cocktails with Cel7B. We showed that while the dissociation rate constant is largely unaffected by the presence of synergy partners, the measured molar association rate constant is an indication of different modes of action of the synergy partner Cel7B. Mainly, the presence of Cel7B led to a reduction of the apparent molar association rate constant, which is consistent with the formation of clusters recently described using high-speed AFM. Furthermore, we demonstrated that increasing the bleaching of the fluorophore during imaging also affects the molar association rate constant in a manner that aligns with the presence of clusters in the Cel7A:Cel7B mixture. We anticipate that our single-molecule assay can lead to further mechanistic understanding of other synergistic systems. In particular, using single-dye labeling of synergy partners with different fluorophores could prove useful.

## Supporting Information Available

Experimental procedures and characterization data for cellulases and of bleaching of Cy5 (PDF)
