# Supplementary material for: Impact of Synergy Partner Cel7B on Cel7A Binding Rates: Insights from Single-Molecule Data
Source: J Phys Chem B. 2024 Jan 16;128(3):635–47. doi: 10.1021/acs.jpcb.3c05697 (PMC10824242; doi:10.1021/acs.jpcb.3c05697)
Supplement: Supplementary file 1 — jp3c05697_si_001.pdf [file jp3c05697_si_001.pdf]

Impact of Synergy Partner Cel7B on Cel7A Binding Rates: Insights from Single-Molecule Data.

Aimilia Nousi,† Gustavo Avelar Molina,‡,§ Corinna Schiano-di-Cola,¶ Trine Holst Sørensen,¶ Kim Borch,¶ Jonas N. Pedersen,† Peter Westh,*,‡ and Rodolphe Marie*,†

†*Department of Health Technology, Technical University of Denmark, 2800 Kongens Lyngby, Denmark*

‡*Department of Biotechnology and Biomedicine, Technical University of Denmark, 2800 Kongens Lyngby, Denmark*

¶*Novozymes A/S, Krogshøjvej 36, DK-2880 Bagsværd, Denmark*

§*Novo Nordisk Foundation Center for Biosustainability, Technical University of Denmark, 2800 Kongens Lyngby, Denmark*

E-mail: PW:pewe@dtu.dk; R.M:rcwm@dtu.dk

Cellulases labeling and purification

The cysteine modification allowed for the specific conjugation of the enzymes with a single organic fluorophore therefore ensuring a single-molecule imaging regime. We chose two fluorophores among the most hydrophilic ones in order to avoid interaction with the purification column, namely maleimide sulfo-Cy5[1] and Alexa 568[2]. All mutant enzymes were labeled with both fluorophores in an effort to determine the best degree of labeling (DOL) while

maintaining the maximum enzyme activity. Purification and separation of free fluorophores and enzyme-conjugated fluorophores was performed using size exclusion chromatography (ÄKTA) and activity measurements were performed after purification. The protocols are described in the Methods section of the main text. Table S1 shows the results of the degree of labeling (DOL) and enzyme activity for the mutant variants of Cel7A and Cel7B labeled.

The *Tr*Cel7A conjugates exhibited low DOLs with both fluorophores as well as a lowered activity compared to the non-labeled enzymes. One hypothesis is that the fluorophore and/or the labeling procedure lower the enzyme activity. Mock labeling experiments using wild-type enzymes subjected to the same labeling procedure revealed however that it was during the purification step of the process that the activity of the enzymes was diminished. Likely, the enzyme samples had a labeled population that was badly folded and did not interact with the purification column. During purification, the healthy enzyme population interacted with it and eluted later at a time point, that would be proportional to their affinity to the matrix material. As a consequence, the fraction of enzymes eluted is the damaged enzyme fraction of lower activity and the active subpopulation that remained bound to the column.

To address this, we chose to label and repurify the Cel7A-T350C mutant with Cy5. However this time, purification was performed with adhesion of the enzyme to cellulose[3]. This separated the labeled enzyme and free fluorophore and preserved the activity of the enzyme compared to the non-labeled enzyme.

Cy5 bleaching time analysis and imaging buffer

Streptavidin labeled with sulfo Cy5 (Lumiprobe, product number: 2449) was immobilized on a glass surface coated with biotinylated Bovine Serum Albumin (BSA) and imaged with TIRF under the same experimental conditions as described in the main text. In order to evaluate the decay time of sulfo Cy5, a stepwise bleaching analysis[4] of the data acquired with this assay was performed.

For this reason, an 8-well glass-bottomed slide (Ibidi) was cleaned to remove autofluorescing impurities with the protocol by Snaar-Jagalska et. al.[5] as described in the main text. The slides were dried under nitrogen flow. Next, 10 µg/mL of BSA were mixed with 10 µg/mL BSA-biotin in a ratio 100:1 and 300 µL of the solution were added to each well and left to incubate for 20 minutes. After that, the solution was removed and the wells were rinsed with 1x Tris-EDTA buffer pH=8. Next, 0.5 µg/mL streptavidin was added and left to incubate for 10 minutes. The solution was then removed and the wells were washed again with 1xTE. Lastly, 0.05 µg/mL sulfo Cy5-biotin was added to the wells and left to incubate for 15 minutes. Afterwards, the solution was removed and the wells were washed again with 1xTE to remove molecules that were not bound. Imaging was done immediately and the unused wells contained 1xTE buffer so the proteins would not dry out. The sulfo Cy5-biotin concentration and incubation time were adjusted so that the molecules appear as isolated bright spots on the surface. The same imaging buffer as in the main cellulases experiments was used, namely a combination of glucose oxidase (10 nM), catalase (0.15 µM), Trolox (1mM) and glucose (55 mM) in 50 mM degassed sodium acetate, pH=5. Nitrogen flow was also used to prevent molecular oxygen from diffusing back into the solution.

Imaging was performed as described in the main text. Three consecutive time-lapse movies of 1000 frames were acquired, each at a different FOV, as was done for the cellulases experiments. Fresh imaging buffer was prepared for each experiment.

After image acquisition, the TrackMate plugin of ImageJ/Fiji was used to detect and record the positions of the biotin-sulfo-Cy5 molecules. The time-lapse movies were binned four times to minimize computation time. The positions of the molecules were then loaded into a custom-made Matlab-based step-finding algorithm[6]. The (x, y) position of each fluorophore is detected in the first frame of every movie through TrackMate and loaded in Matlab. Then the script searches the raw images for those coordinates and sums the intensity of a 5x5 pixel area around the spot (Fig. S9a). The intensity is then plotted over time (Fig. S9b). The algorithm utilizes a maximum likelihood estimator to fit the time trace

and find the minimum number of bleaching steps (i.e. the number of fluorophores present in the spot). The step size of a single fluorophore decay as well as the timepoint of each fluorophore decay (Fig. S9c) are determined. A range of step sizes is given as input to the algorithm, in order to start the search. With this, we were able to generate the bleaching time of each Cy5 molecule in a number of spots. After the fitting was performed, the generated fits and durations were visually inspected, and obvious outliers were removed. After the acquisition of the lifetimes, they were converted to physical units (seconds) and fitted with double exponential decays. The two acquired characteristic times are shown in Fig. S10.

Single molecule image analysis using Trackmate

We use Trackmate to extract the residence time of spots in the time-lapse movies. In this work, the LoG detector was used. It applies a Laplacian of Gaussian filter on the image and detects the local maxima in the filtered image. An important filtering step is the "Estimated object diameter", here set to 0.6 µm in order to account for possible drift of the focus of the microscope in the vertical direction (z) that increases the width of the diffraction-limited spots. Moreover, the "Quality Threshold" was set to 300 to exclude the detection of background maxima. Visual inspection confirmed that this gave the best results for the detection of actual single enzyme spots with minimal noise included. Next, the actual tracking of the spots i.e. linking of the spots detected in consecutive frames into continuous tracks is performed. The simple Linear Assignment Problem (LAP) tracker algorithm was used. In our data, a track might be interrupted due to blinking of the fluorophore or the spot failing to be detected in a frame due to a low signal-to-noise ratio since we use a low excitation to extend the lifetime of the fluorophore. Trackmate deals with those missing frames by introducing two parameters that can be set by the user, "Gap-closing max distance" and "Gap-closing max frame gap". Then the algorithm will look in the next frames and further

away for the missing spots in order to link a candidate spot to the existing track. After a manual inspection of individual tracks, the parameters were set to a linking max distance of 0.09 µm, a gap-closing max distance of 0.2 µm, and a gap-closing max frame gap of 5. Those parameters made the track detection robust, in particular to drift of the focus. Table S2 provides a summary of the parameters mentioned above. Note that the linking distance is small compared to the diffraction limit because we do not expect the cellulases to move significantly ($\approx 3\,\mathrm{nm/s}$[7,8]) during the interval between two consecutive frames (2 seconds).

Next, before finalizing the tracks, the user can define another set of filters to further improve the results of the tracking. Here, we chose that the total number of spots in a track should be more than 5, i.e. a track duration of at least 10 seconds, in order to be considered an event. Manual inspection of individual tracks followed by manual gap closing was performed on several movies to ensure that the residence time measurement was not biased by the choice of parameters.

The track and spot information such as position (x, y, t), the mean intensity of each spot as well as start- and end-time of the tracks were exported and then further processed using a custom Matlab analysis script.

The first step in the analysis is to discard tracks that 1) were already present on the first frame of the time-lapse movie or 2) did not end before the end of the time-lapse movie and were rejected from the analysis. This is done to ensure that the length of a track actually represents the actual enzyme residence time.

Furthermore, we need to account for artefacts at the beginning of the second and third time-lapse movies of each experiment. Those two time-lapses are recorded at new positions on the substrate after 40 and 80 minutes of exposure to enzymes without laser excitation. Consequently, there are many spots on the first frames corresponding to 'stuck' enzymes with an active fluorophore. In those spots, enzymes that are very close to each other, as one fluorophore bleaches out, neighbor enzymes are detected as new 'tracks' by our automatic analysis in Trackmate. Therefore, for every movie, the number of new tracks in each frame

was plotted over time in order to estimate when a steady-state enzyme arrival rate was reached. Tracks that started before that frame were all rejected.

Finally, the histograms of the durations of the tracks were fitted using maximum likelihood estimation with double-exponential distributions to extract the spot lifetime as the fitted parameter of the shortest exponential decay.

Calculations of $k_{\mathbf{OFF}}$ values from the spot lifetime

The measured lifetime of a spot, t_{meas} is the minimum of two events: the bleaching of the fluorophore t_{bleach} and the enzyme unbinding t_{res}. Both t_{bleach} and t_{res} are assumed to be exponentially distributed with characteristic timescales τ_{bleach} and τ_{res} respectively.

That is:

$$p(t_{\text{bleach}}) = (1/\tau_{\text{bleach}}) \cdot exp(-t_{\text{bleach}}/\tau_{\text{bleach}}) \tag{S1}$$

and

$$p(t_{\text{res}}) = (1/\tau_{\text{res}}) \cdot exp(-t_{\text{res}}/\tau_{\text{res}}) \tag{S2}$$

As mentioned, t_{meas} is the minimum of t_{bleach} and t_{res}, i.e. $t_{\text{meas}} = \min(t_{\text{bleach}}, t_{\text{res}})$ From statistics, it is known that the minimum of two independent, exponentially distributed variables will also be exponentially distributed. The inverse of the characteristic scale is the sum of the inverse scales of the two distributions:

$$1/\tau_{\text{meas}} = 1/\tau_{\text{bleach}} + 1/\tau_{\text{res}} \tag{S3}$$

with τ_{meas} the measured spot lifetime, τ_{bleach} the characteristic bleaching time of the fluorophore in the imaging conditions, and τ_{res} the residence time of the enzyme. We then

calculate the residence time (and the k_{OFF}) using:

$$k_{\mathrm{OFF}} = 1/\tau_{\mathrm{res}} = 1/\tau_{\mathrm{meas}} - 1/\tau_{\mathrm{bleach}}. \tag{S4}$$

Notice that, in the limit $\tau_{\mathrm{bleach}} \gg \tau_{\mathrm{res}}$ we find that $\tau_{\mathrm{res}} \sim \tau_{\mathrm{meas}}$.

In our example for Cel7A-Cy5 we measure τ_{meas} =46 s and find from the calibration, using τ_{bleach}=110 s, that a spot lifetime of 46 s corresponds to τ_{res} =79 s, and k_{OFF} is 0.013 s^{-1}.

Error propagation: We use the covariance matrix of the fit of the lifetime histograms to get the error on τ_{meas} and on τ_{bleach}.

To calculate the error propagation on τ_{res} we use Eq (3.18) in Taylor[9] and write:

$$\tau_{\mathrm{res}} = \frac{\tau_{\mathrm{meas}}\tau_{\mathrm{bleach}}}{\tau_{\mathrm{bleach}} - \tau_{\mathrm{meas}}} \tag{S5}$$

We then get that the fractional uncertainty on τ_{res} is:

$$\frac{\sigma_{\mathrm{res}}}{\tau_{\mathrm{res}}} = \sqrt{(\frac{\sigma_{\mathrm{meas}}}{\tau_{\mathrm{meas}}})^2 + (\frac{\sigma_{\mathrm{bleach}}}{\tau_{\mathrm{bleach}}})^2 + (\frac{\sqrt{\sigma_{\mathrm{meas}}^2 + \sigma_{\mathrm{bleach}}^2}}{\tau_{\mathrm{bleach}} - \tau_{\mathrm{meas}}})^2} \tag{S6}$$

References

(1) Mudinoor, A. R.; Goodwin, P. M.; Rao, R. U.; Karuna, N.; Hitomi, A.; Nill, J.; Jeoh, T. Interfacial molecular interactions of cellobiohydrolase Cel7A and its variants on cellulose. *Biotechnology for Biofuels* **2020**, *13*, 10.

(2) Wang, L.; Wang, Y.; Ragauskas, A. J. Determination of cellulase colocalization on cellulose fiber with quantitative FRET measured by acceptor photobleaching and spectrally unmixing fluorescence microscopy. *Analyst* **2012**, *137*, 1319–1324.

(3) Moran-Mirabal, J. M.; Corgie, S. C.; Bolewski, J. C.; Smith, H. M.; Cipriany, B. R.;

Craighead, H. G.; Walker, L. P. Labeling and purification of cellulose-binding proteins for high resolution fluorescence applications. *Analytical chemistry* **2009**, *81*, 7981–7987.

(4) Hundahl, A. C.; Weller, A.; Larsen, J. B.; Hjørringgaard, C. U.; Hansen, M. B.; Mündler, A.-K.; Knuhtsen, A.; Kristensen, K.; Arnspang, E. C.; Andresen, T. L., et al. Quantitative live-cell imaging of lipidated peptide transport through an epithelial cell layer. *Journal of Controlled Release* **2023**, *355*, 122–134.

(5) Snaar-Jagalska, B. E.; Cambi, A.; Schmidt, T.; Keijzer, S. D. *Single-molecule imaging technique to study the dynamic regulation of gpcr function at the plasma membrane*, 1st ed.; Elsevier Inc., 2013; Vol. 521; pp 47–67.

(6) Ulbrich, M. H.; Isacoff, E. Y. Subunit counting in membrane-bound proteins. *Nature methods* **2007**, *4*, 319–321.

(7) Haviland, Z. K.; Nong, D.; Kuntz, K. L.; Starr, T. J.; Ma, D.; Tien, M.; Anderson, C. T.; Hancock, W. O. Nanoscale dynamics of cellulose digestion by the cellobiohydrolase Tr-Cel7A. *Journal of Biological Chemistry* **2021**, *297*, 101029.

(8) Igarashi, K.; Koivula, A.; Wada, M.; Kimura, S.; Penttilä, M.; Samejima, M. High speed atomic force microscopy visualizes processive movement of Trichoderma reesei cellobiohydrolase I on crystalline cellulose. *Journal of biological chemistry* **2009**, *284*, 36186–36190.

(9) Taylor, J. Introduction to Error Analysis, the Study of Uncertainties in Physical Measurements. *Published by University Science Books* **1997**, 45–91.

Supplementary tables and figures.

List of Tables

S1 DOLs and activity measurements S10

S2 Parameters for TrackMate analysis S10

List of Figures

S1 Kinetic parameters of the cysteine variants. S11

S2 Activity measurements. S12

S3 Length of cellulose fibers per FOV. S13

S4 Home-built TIRF set-up. S14

S5 SDS-PAGE analysis of the cysteine variants. S15

S6 Cysteine variants of Cel7A S16

S7 Cysteine variants of Cel7A S17

S8 Mock labeling procedure of WT Cel7A S17

S9 Cy5 photo-bleaching data example. S18

S10 Photo-bleaching of Cy5 at $12\,W/cm^2$. S18

S11 Residence time correction with the Cy5 photo-bleaching time at increasing illumination power density. S19

Table S1: DOLs and activity measurements (% compared to non-labeled) of the labeled enzymes expressed and purified for this study.

Variant	Fluorophore	DOL (%)	Activity (%)
*Tr*Cel7A-T350C	Alexa 568	27.4	8
*Tr*Cel7A-T350C	Sulfo-Cy5	28.6	8
*Tr*Cel7A-T350C	Sulfo-Cy5	28	116
*Tr*Cel7A-S87C	Alexa 568	34	16
*Tr*Cel7A-S87C	Sulfo-Cy5	31.4	20
*Tr*Cel7B-A303C	Alexa-568	101	110
*Tr*Cel7B-A303C	Sulfo-Cy5	118	100

Table S2: Parameters used for tracking of the *Tr*Cel7A molecules in Trackmate.

Estimated object diameter	0.6 µm
Quality Threshold	300
Linking max distance	0.09 µm
Gap-closing max distance	0.2 µm
Gap-closing max frame gap	5

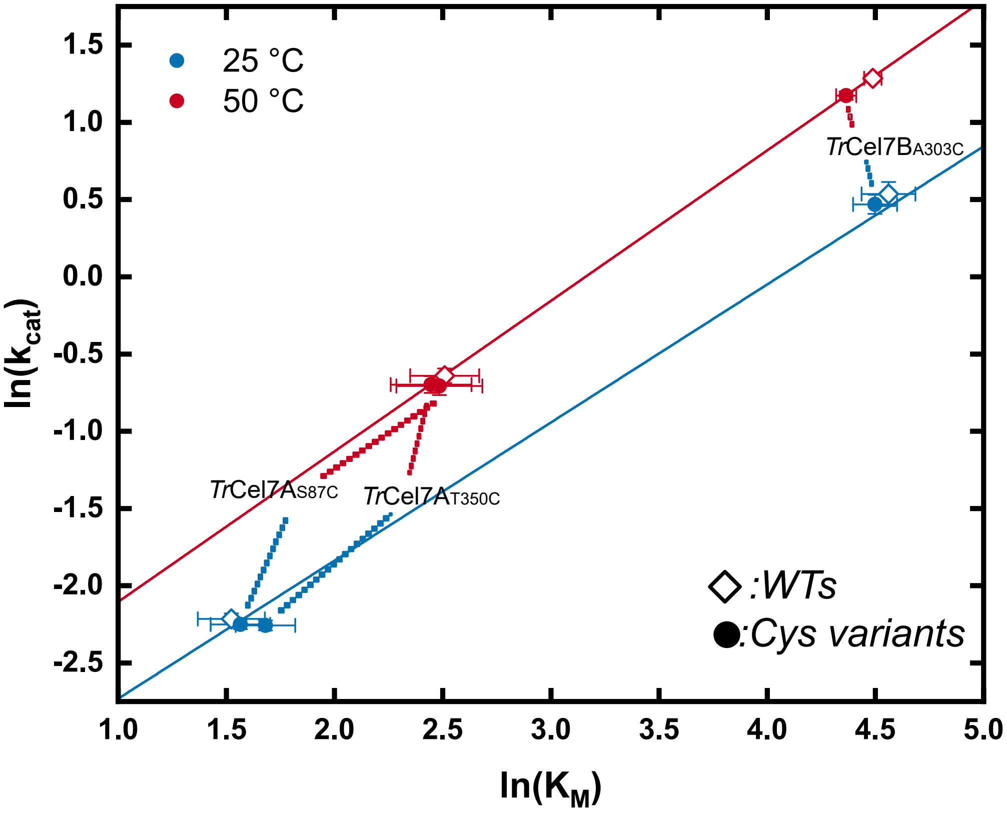

Figure S1: **Kinetic parameters of the cysteine variants and their respective WTs.** Characterization of the cysteine variants plus their respective WTs (wild types) against Avicel at MM condition and 25 and 50 °C. The kinetic parameters of all cysteine variants are essentially the same as the WTs at the two temperatures.

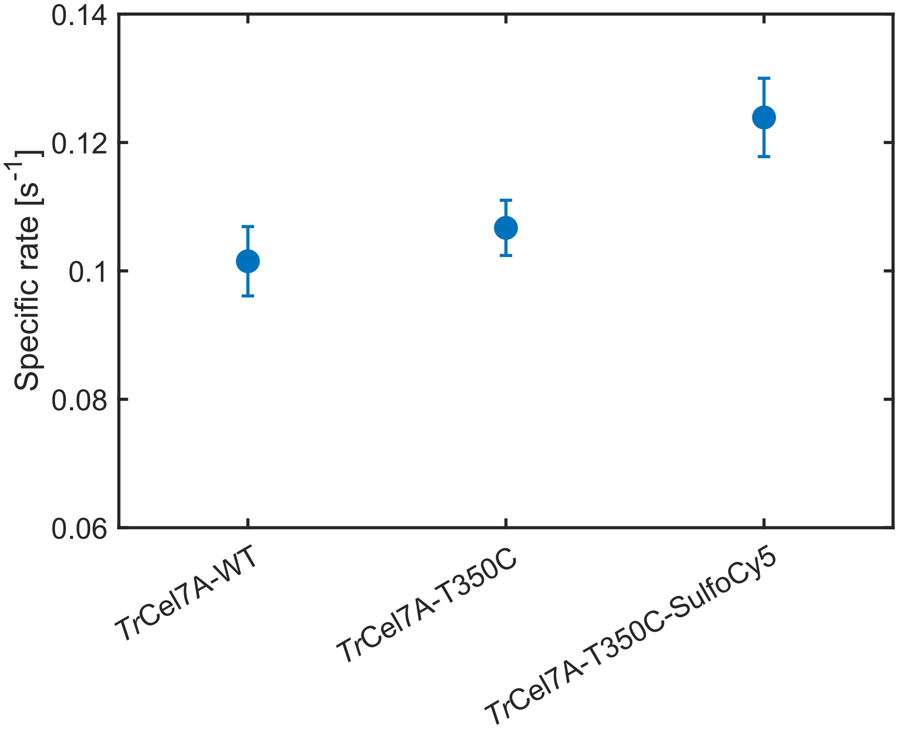

Figure S2: **Activity measurements of *Tr*Cel7A-WT and mutant.** The activity of the labeled mutant *Tr*Cel7A-T350C-SulfoCy5 is not affected by the labeling procedure. The error bars are the standard deviation of six measurements.

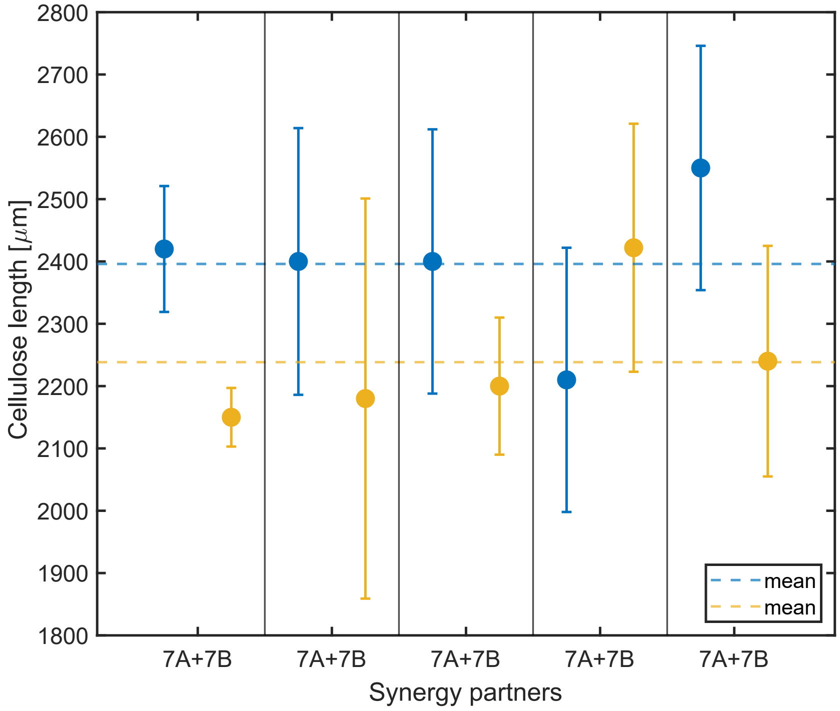

Figure S3: **Cellulose lengths per field of view** measured on bright field images using a skeletonization algorithm in ImageJ/FIJI. Each data point corresponds to the mean of three field-of-view images, one for each fluorescence time-lapse movie recorded at a given enzymatic composition. The data correspond to five paired experiments displayed in main text Figure 3c that are replicas comparing Cel7A and Cel7A:Cel7B.

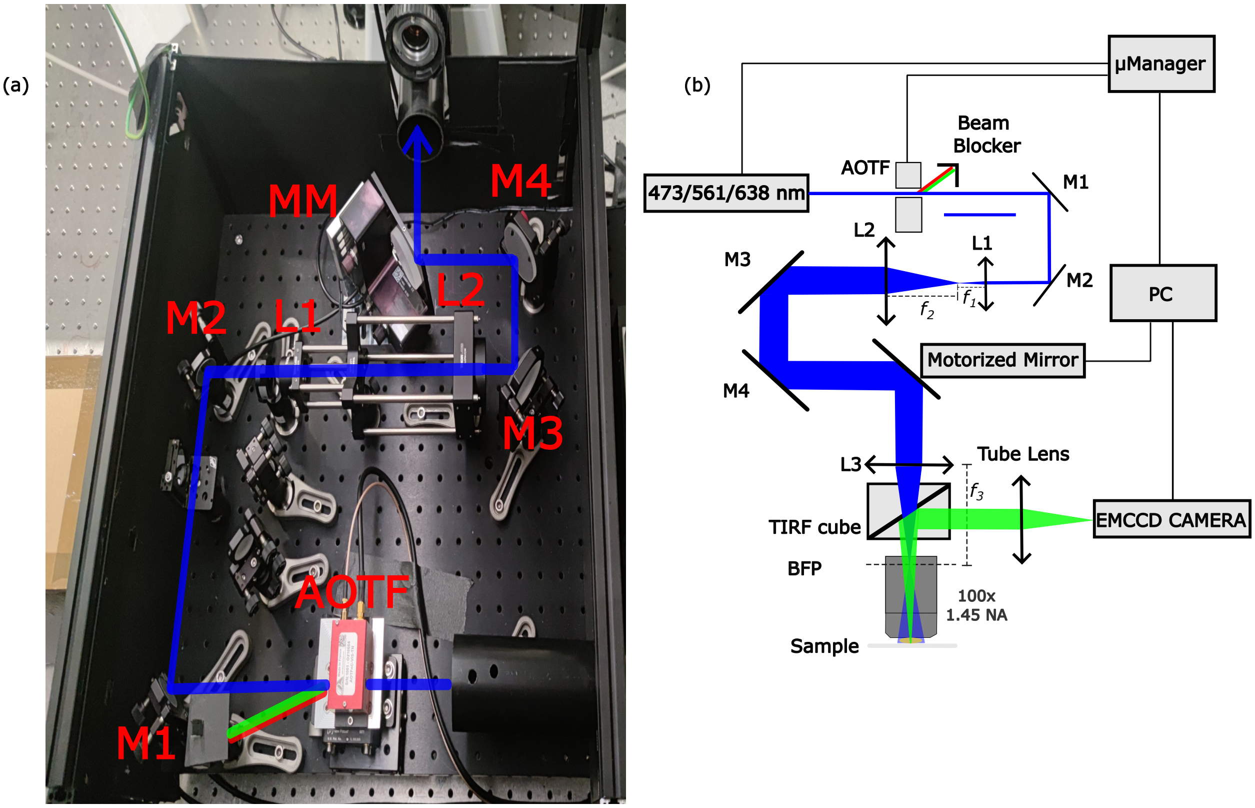

Figure S4: **Home-built TIRF set-up** including a three-color combined laser source (Cobolt, C-Flex), an AOTF (AA Optoelectronics), a telescope (L1 and L2), a motorized mirror (Zaber) placed in a 4-f configuration with the projecting lens (L3, f_3=300 mm) and the objective (Nikon lambda 100x/1.45).

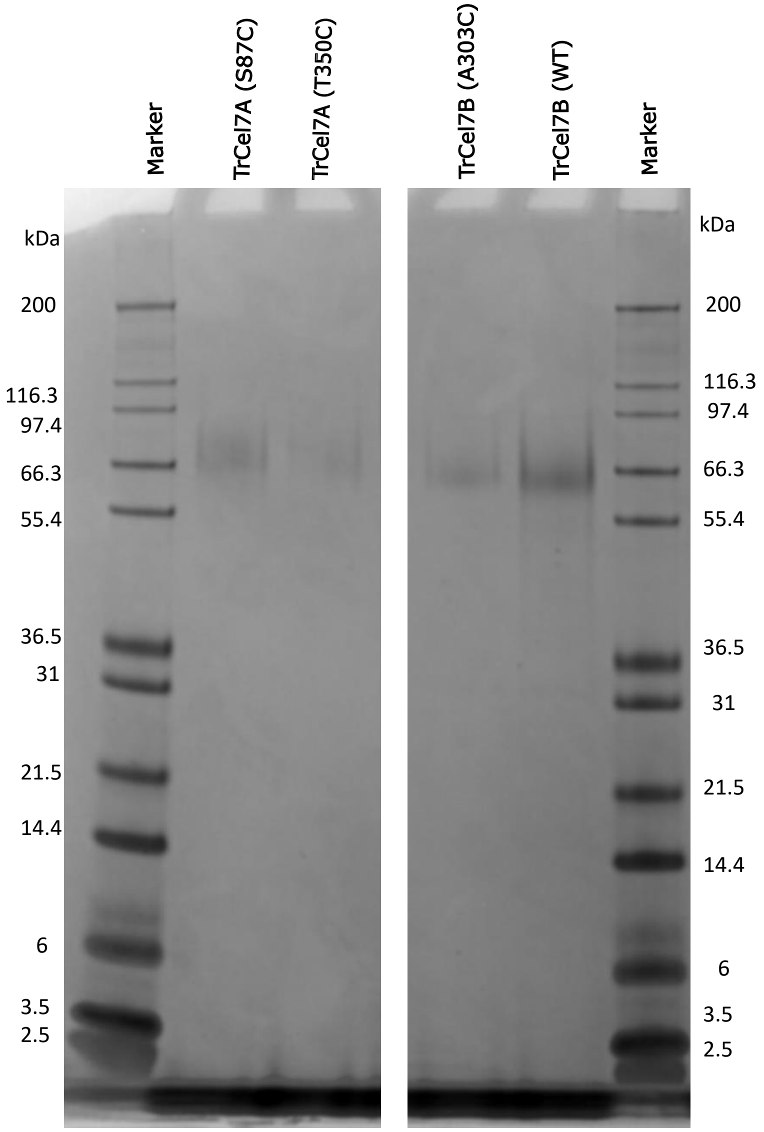

Figure S5: **SDS-PAGE analysis of the cysteine variants.** Lane 1 and 6: Marker Mark12 (Thermo Fisher) molecular weight standard. Lane 2: Cel7A cysteine mutant, S87C mutation. Lane 3: Cel7A cysteine mutant, T350C mutation. Lane 4: Cel7B cysteine mutant, A303C mutation. Lane 5: Cel7B WT. The gel was stained with Coomassie Blue and 1 µg of protein was loaded onto the gel.

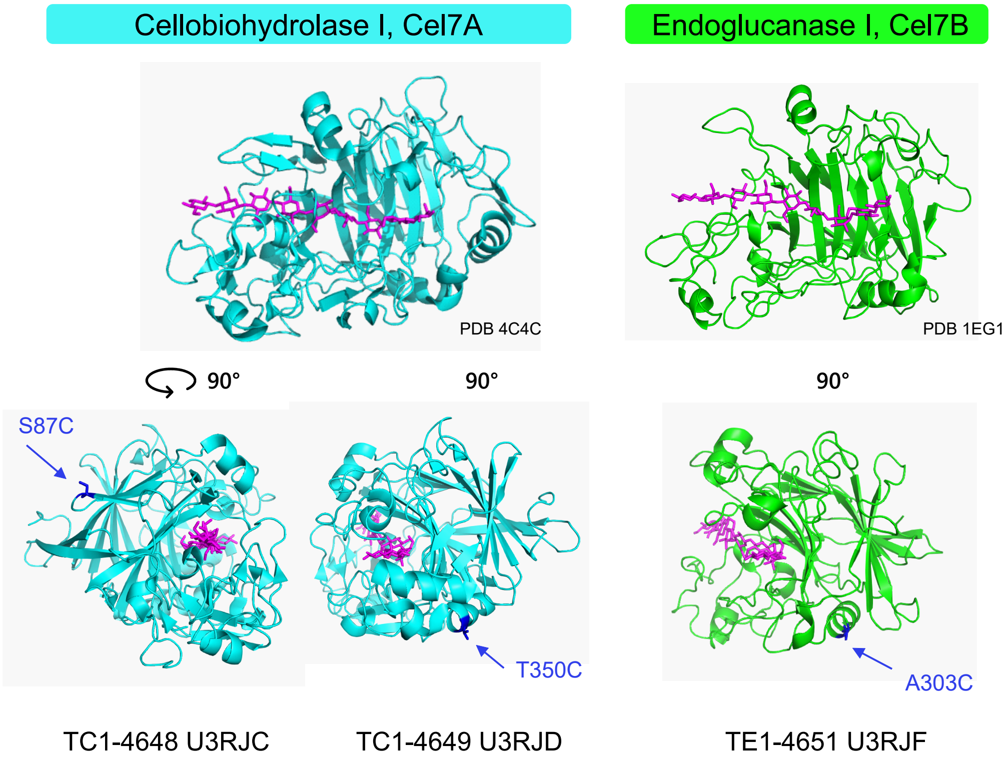

Figure S6: **Overview of cysteine variants** of the cellulases from *Trichoderma reesei*.

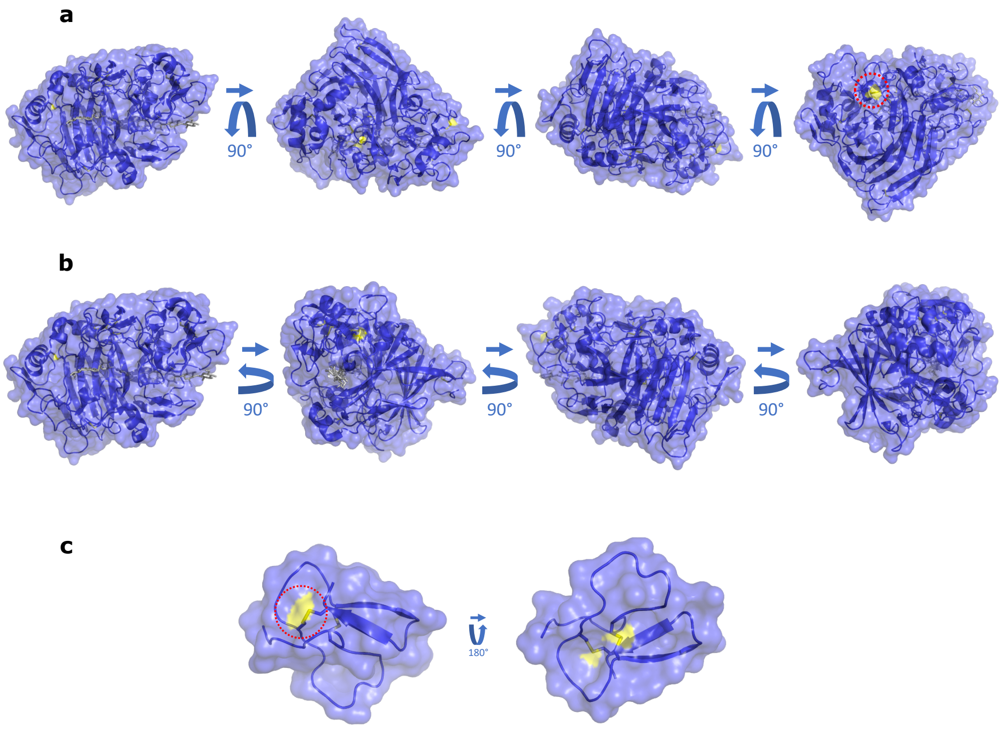

Figure S7: **Structural representation** of *Tr*Cel7A core (a and b) (PDB ID: 4C4C), where the sulfur atoms of the cysteines are highlighted in yellow, and the red dashed circle emphasizes the cysteines with the highest probability of suffering unintentional labeling. The last panel is a schematic of the CBM of *Tr*Cel7A (PDB ID: 1AZ6). Images made in PyMOL.

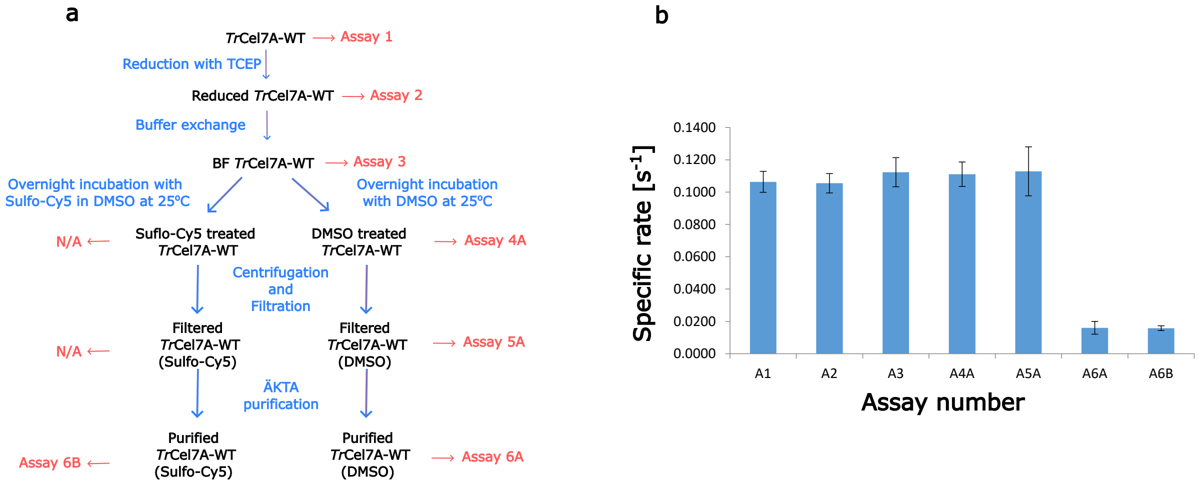

Figure S8: **Mock labeling procedure of WT Cel7A.** (a) A schematic representation of the steps for the labeling as well as the activity assays. (b) Activities of Cel7A for each step of the procedure. Both DMSO and Sulfo-Cy5 samples lost 85% of activity in relation to sample A1 (stock enzyme). Conditions: 90 g/L of Avicel, 25 C, 1 h, 1100 Rpm.

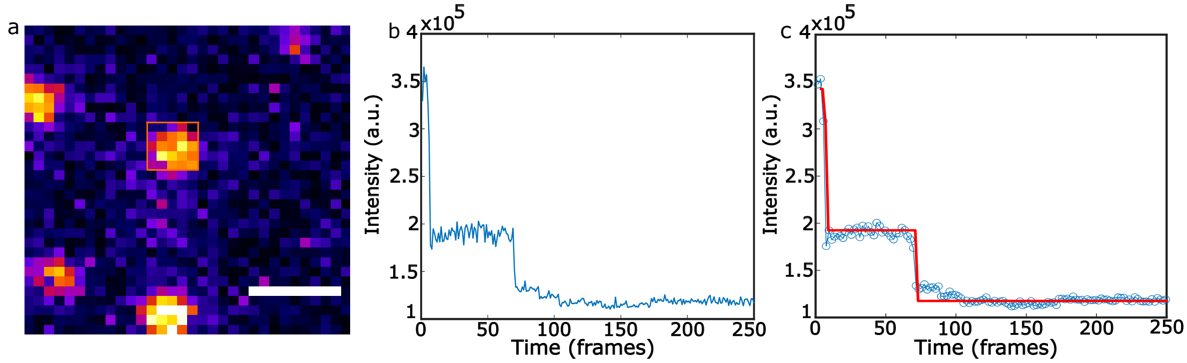

Figure S9: **An example of a biotin-Cy5 conjugate showing photo-bleaching steps.**(a) Single frame raw data with the 5x5 pixel bounding box around a bright spot, were the intensity in the box is summed over time and used for analysis. Scale bar is 1 µm. (b) A typical intensity trace (blue line) as a function of the number of frames for the bio-conjugate shown in (a). The trace shows two photo-bleaching steps. (c) Fit obtained from the step-finding algorithm using the experimental data shown in (b). For this particular spot, two steps were found corresponding to two fluorophores bleaching hence two different bleaching times were measured.

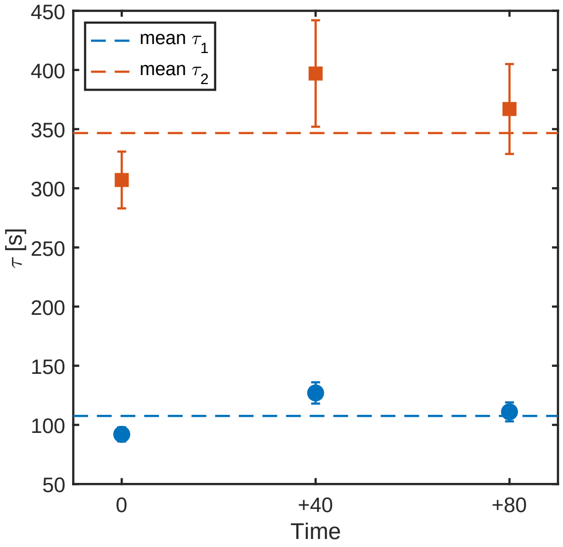

Figure S10: **Photo-bleaching of Cy5 at 12** W/cm^2**.** Characteristic time constants as obtained through double exponential decay fitting with maximum likelihood estimation. The short (τ_1) and long (τ_2) characteristic times of Cy5 bleaching for each imaging time point used t=0, 40 and 80 minutes.

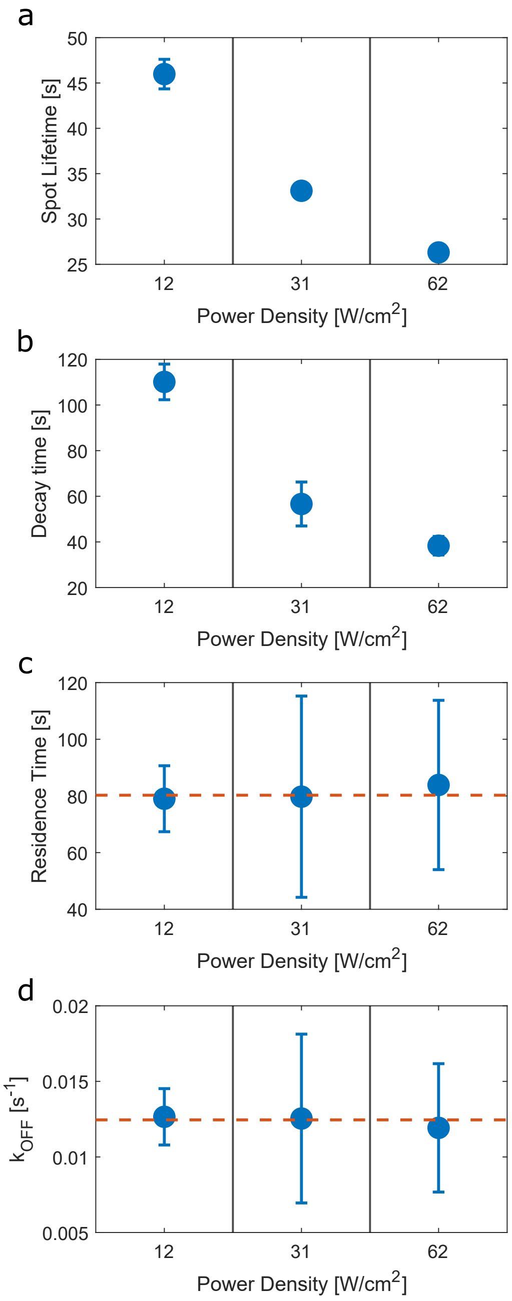

Figure S11: **Residence time correction with the Cy5 photo-bleaching time $\tau_{\mathbf{bleach}}$ at increasing illumination power density.** a) The spot lifetime decreases with illumination power density. b) This is mainly due to the reduced photo-bleaching time of single Cy5 molecules (short τ_{bleach} is shown). c) After correction, the residence time is consistent with a common mean for all three power densities tested. d) This illustrates that the measurement of the k_{OFF} is robust for photo-bleaching if correction is performed.
